# A Potential Four-Gene Signature and Nomogram for Predicting the Overall Survival of Papillary Thyroid Cancer

**DOI:** 10.1155/2022/8735551

**Published:** 2022-08-30

**Authors:** Ying Ding, Peng Li, Wenlong Wang, Fang Liu

**Affiliations:** ^1^Thyroid Surgery Department, Xiangya Hospital, Central South University, Changsha 410008, China; ^2^National Clinical Research Center for Geriatric Disorders, Xiangya Hospital, Central South University, Changsha, 410008 Hunan Province, China; ^3^Health Management Center, Xiangya Hospital, Central South University, Changsha 410008, China

## Abstract

**Background:**

Although the prognosis of papillary thyroid cancer (PTC) is relatively good, some patients experience recurrence or distant metastasis after thyroidectomy and progress to radioactive iodine refractory stage. Therefore, accurate prediction of clinical outlook can aid to screen out the minority of patients with poorer prognosis and avoid excessive treatment in low-risk patients.

**Methods:**

The RNA-seq and clinical data of PTC patients was downloaded from the Gene Expression Omnibus (GEO) and the Cancer Genome Atlas (TCGA) databases. Multivariate and Lasso Cox regression analyses were used to construct a prognostic nomogram to predict overall survival (OS). Thereafter, quantitative RT-PCR and Human Protein Atlas (HPA) database were employed to verify the expression of key genes.

**Results:**

A four-gene risk score comprising *ABI3BP*, *DPT*, *MRO*, and *TENM1* was exhibited strong prognostic value. Moreover, an integrated nomogram was established based on the risk score, age, AJCC (American Joint Commission on Cancer) stage, tumor size, extrathyroidal extension, and history of neoadjuvant treatment, which exhibited significantly better predictive performance than TNM stage system (*P* < 0.05). GSEA (Gene Set Enrichment Analysis) and GSVA (Gene Set Variation Analysis) revealed that the different tumor-associated hallmarks, biological processes, and pathways were substantially enriched in the poor-prognosis group. In addition, a ceRNA network was constructed by including the four genes (*ABI3BP*, *DPT*, *MRO*, and *TENM1*), 54 lncRNAs, and 10 miRNAs. Finally, both the relative mRNA and protein expression of ABI3BP, DPT, MRO, and TENM1 were validated.

**Conclusion:**

The present study identified a four-gene risk signature and developed a novel nomogram, which could be regarded as a reliable prognostic model for PTC patients. The findings also revealed preliminary potential mechanisms that may influence the prognosis outcome. These results can be conducive to design personalized treatment and prognosis management in affected patients.

## 1. Introduction

In the past few decades, the incidence of thyroid cancer has increased rapidly worldwide [1]. Papillary thyroid carcinoma (PTC) is the most common pathological subtype, which has accounted for more than 85% of cases [2]. PTCs usually presents an excellent prognosis, and 10-year disease-specific survival rates have been reported to be over 90% via management through the common therapeutic approaches such as thyroidectomy, RAI therapy, and thyroid-stimulating hormone (TSH) suppressive therapy [3]. However, local recurrence and distant metastasis inevitably occur in up to 20% and 10% in PTC patients [4]. Moreover, two-thirds of these patients exhibit loss of iodine-131 (131I) uptake initially or gradually, thereby indicating dedifferentiation of the PTC termed as RAI-refractory PTC [5]. Thus, accurate assessment of the prognosis of PTC is critical to ensure that the high-risk patients receive appropriate treatment and to prevent excessive treatment of the low-risk patients.

The 8^th^ edition of the AJCC/TNM (tumor node metastasis) manual was released in 2017 [6, 7]. TNM staging has been identified to be useful in predicting the disease mortality, and it is recommended for all PTC patients [7, 8]. However, it has been difficult to accurately distinguish the difference between the survival outcomes in PTCs with similar clinicopathological features [9, 10]. A number of prior studies have proposed that combining *BRAF*, *TERT*, and *RAS* mutations with TNM staging can lead to better prediction of the prognosis of PTC patients [11–13]. Our team has previously constructed an integrated nomogram based on clinicopathological factors and the related risk scores, which showed significantly better predictive performance than AJCC stage [14–16].

In this study, we aimed to develop a prognosis-predicting model based on nomogram as a prognostic evaluation method. We have obtained data of PTC patients from The Cancer Genome Atlas (TCGA) and Gene Expression Omnibus (GEO) databases. Univariate and multivariate Cox regression analysis and Lasso regression analysis was performed to establish a four-gene signature in the training cohort. Time-dependent receiver-operating characteristic (ROC) and Kaplan-Meier (KM) curve was used to assess the validity of the four-gene signature and the nomogram in the entire patient cohort.

## 2. Materials and Methods

### 2.1. Acquisition and Processing of Data

**Figure 1:**
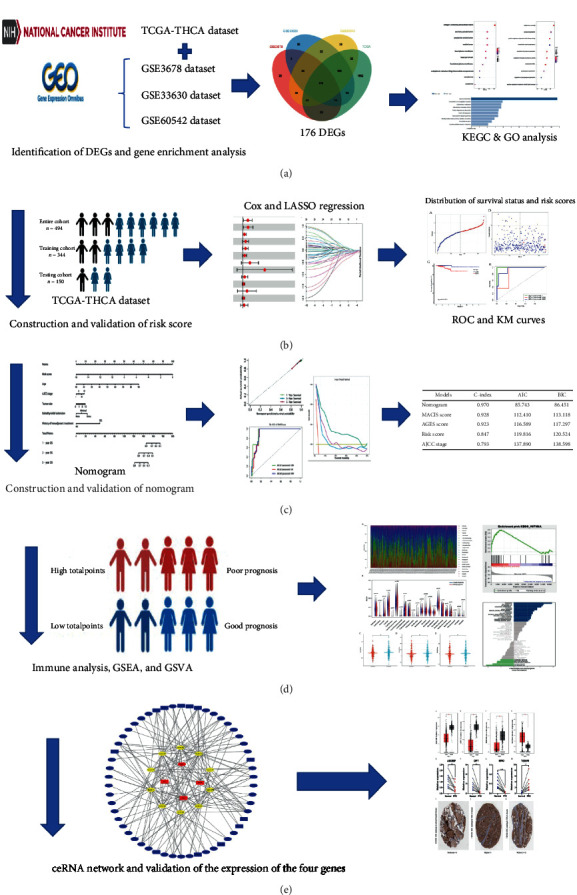
Flowchart of the study design.

ALL RNA-seq data and the clinical characteristics were extracted from three GEO datasets (GSE33630, GSE3678, and GSE60542) and TCGA-THCA dataset (Table S1). A total of 494 PTC samples were selected from the TCGA-THCA dataset and were then enrolled for the subsequent analysis. The flowchart of the designed study has been illustrated in Figure 1.

### 2.2. Screening of the Differentially Expressed Genes and Gene Enrichment Analysis

Differentially expressed genes (DEGs) between the normal and PTC samples with absolute log2 fold change (FC) > 1.5 and *P* < 0.05 were screened from three GEO datasets and the TCGA-THCA dataset by using the “limma” R package. Enrichment analyses of Gene Ontology (GO) and Kyoto Encyclopedia of Genes and Genomes (KEGG) pathway for DEGs were performed by using the “clusterProfiler” R package and Webgestalt website (http://www.webgestalt.org/).

### 2.3. Establishment and Validation of the Four-Gene Risk Signature

In total, 344 samples were randomly selected from the 494 PTC samples in the TCGA-THCA dataset as the training cohort. The remaining 150 samples were used as the testing cohort. In the training cohort, 35 OS-related genes were selected based on univariate cox analysis. Thereafter, least absolute shrinkage and selection operator (LASSO) regression was used to screen 12 potential prognostic genes. Finally, only 4 genes (*P* < 0.05) were included in the risk signature based on the results of multivariate Cox regression. Finally, the risk score for each patient was calculated as follows: ∑_*i*=1_^*n*^coef(*i*)∗exp(*i*), where exp(*i*) represents the expression of genes, and coef(*i*) is the coefficient of multivariate Cox regression. ROC and KM curves for risk score were drawn with the “survival” and “timeROC” R packages to assess the potential predictive capacity of the risk signature in the training cohort, testing cohort, and the entire cohort.

### 2.4. Construction and Evaluation of the Nomogram

Univariate and multivariate Cox regression analyses were utilized to screen the various essential clinical characteristics related to OS. A nomogram was established based on the risk score, age, AJCC stage, tumor size, extrathyroidal extension, and history of neoadjuvant treatment with the “rms” R package. The *C*-index, Akaike information criterion (AIC), and Bayesian Information Criterion (BIC) of the nomogram were calculated, and the ROC, KM, calibration, and decision curves were drawn.

### 2.5. Immune Analysis

The median nomogram point was used to divide the entire cohort into two distinct groups of good-prognosis and poor-prognosis. Cell-type Identification By Estimating Relative Subsets Of RNA Transcripts (CIBERSORTx) (https://cibersortx.stanford.edu/) was then employed to analyze the immune infiltration of 22 types of immune cells in good-prognosis and poor-prognosis groups. The stroma and immune scores were measured by Estimation of Stromal and Immune cells in Malignant Tumor tissues using Expression data (ESTIMATE) analysis using “estimate” R package.

### 2.6. Gene Set Enrichment Analysis (GSEA) and Gene Set Variation Analysis (GSVA)

GSEA and GSVA were performed with the GSEA software and the “GSVA” R package. The reference gene sets in GSEA were the c2.cp.kegg.v7.5.1.symbols.gmt, and if the normal *P* value < 0.05 and FDR (false discovery rate) *q* value < 0.25, the gene set was considered as significantly enriched.

### 2.7. Construction of a Competing Endogenous RNA Regulatory Network

The (differentially expressed miRNAs) DEmiRNAs and (differentially expressed lncRNAs) DElncRNAs between the normal and PTC samples in the TCGA-THCA dataset with absolute log2 fold change (FC) > 1.5 and *P* < 0.05 were screened by using the “limma” R package. The miRcode database was used to match DElncRNAs and DEmiRNAs. ABI3BP, DPT, MRO, and TENM1 target miRNAs were predicted based on the three distinct databases: miRWalk, TargetScan, and miRmap. Subsequently, a ceRNA regulatory network was constructed according to the results obtained above. Cytoscape (version 3.8.2) was used to visualize the competing endogenous RNA (ceRNA) network. OS-related DEmiRNAs and DElncRNAs were screened by the KM curves.

### 2.8. Data Mining in the GEPIA and HPA Databases

The RNA expression data of *ABI3BP*, DPT, MRO, and TENM1 genes in normal and PTC samples were extracted from the Gene Expression Profiling Interactive Analysis (GEPIA) database. The immunohistochemistry (IHC) data was obtained from seven patients in the Human Protein Atlas (HPA) database, for whom the basic information is available as shown in Table S2.

### 2.9. Patients

Ten paired PTC tumors and adjacent normal thyroid tissues were obtained from patients in the Thyroid Surgery Department of Xiangya Hospital from March 2020 to June 2020. An informed consent was obtained from all the participants, and the study was approved by the Ethics Committee of Xiangya Hospital of Central South University (No. 202004192).

### 2.10. Quantitative RT-PCR

Total RNA was extracted from the normal and PTC tissues by using Trizol Reagent (Accurate Biology, China). cDNA synthesis was performed using Reverse Transcription Kit (Accurate Biology, China). Quantitative RT-PCR was then carried out with the RT-PCR Kit (Accurate Biology, China). The sequence of all the primers used have been described in Table S3.

### 2.11. Statistical Analysis

Statistical analysis was performed based on R software (version 4.1.0). Chi-square test or Fisher's exact test were used to analyze the categorical variables. *T*-test and one-way ANOVA were used to analyze the continuous variables. Univariate and multivariate Cox regression and log-rank test were performed to evaluate OS. Unless otherwise stated, *P* < 0.05 indicated that the difference was statistically significant.

## 3. Results

### 3.1. Identification of DEGs and Gene Enrichment Analysis

**Figure 2:**
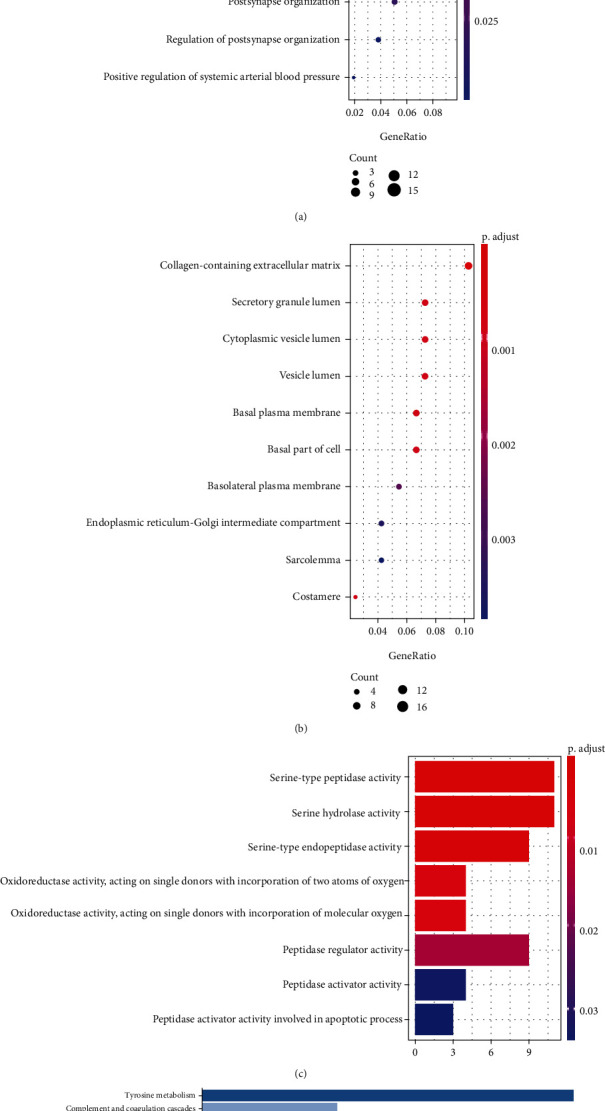
Gene enrichment analysis. (a–c) Top 10 enriched biological processes (BP), cellular components (CC), and molecular functions (MF) of the DEGs. (d) Top 10 KEGG pathways were obtained by enrichment analysis.

The value distribution of the selected samples in the three GEO datasets is uniform which has been shown in Supplementary Figure 1. The various DEGs were obtained in three GEO datasets and TCGA-THCA datasets (Supplementary Figure 2A-D) (453 in GSE33630, 323 in GSE3678, 442 in GSE60542, and 2328 in the TCGA-THCA dataset). The intersection of the four datasets contained 176 DEGs (Supplementary Figure 2E), including 102 upregulated genes and 74 downregulated genes. GO analysis demonstrated that these 176 DEGs were primarily enriched in cell junction assembly, synapse organization, and positive regulation of protein serine in the biological process, collagen-containing extracellular matrix, secretory granule lumen, and cytoplasmic vesicle lumen in the cellular components, serine-type peptidase activity, serine hydrolase activity, and serine-type endopeptidase activity in the molecular functions (Figures 2(a)–2(c)). Additionally, KEGG pathway enrichment analysis indicated that DEGs were significantly enriched in tyrosine metabolism (Figure 2(d)). These results suggested that the genesis and development of PTC might be closely related to these genes or pathways, including pathways that play an important role in development of cancers, such as serine activity, tyrosine metabolism, extracellular matrix, and cellular junction. Therefore, these results can guide our subsequent study related to understanding the molecular mechanisms of PTC.

### 3.2. Establishment of the Four-Gene Risk Signature

**Figure 3:**
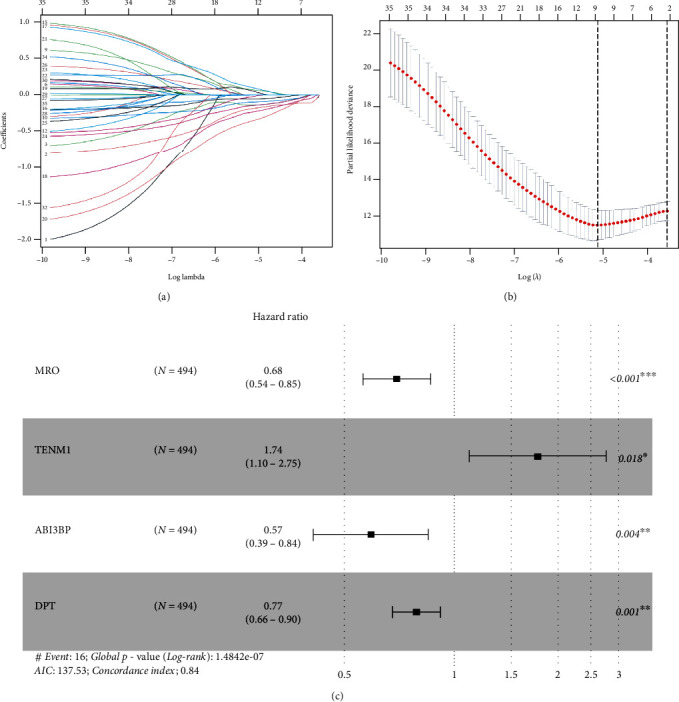
Establishment of the four-gene signature. (a, b) 12 genes were screened by Lasso regression analysis of the 35 OS-related genes. (c) The final four key genes were selected by performing multivariate Cox regression of the 12 genes.

The baseline of the clinical characteristics of the training cohort, testing cohort, and the entire cohort has been presented in Table 1. 35 genes (*P* < 0.05) related to OS were screened by univariate Cox regression in the training cohort (Table S4). After performing Lasso regression analysis on these 35 genes, 12 genes were obtained (Figures 3(a) and 3(b)). Finally, 12 genes were analyzed by using multivariate Cox regression, and *ABI3BP*, *DPT*, *MRO*, and *TENM1* were selected to construct the four-gene risk signature (Figure 3(c)). The risk score = −0.56124 × ABI3BP + (−0.25805 × DPT) + (−0.38946 × MRO) + 0.55421 × TENM1.

### 3.3. Validation of the Four-Gene Risk Signature

**Figure 4:**
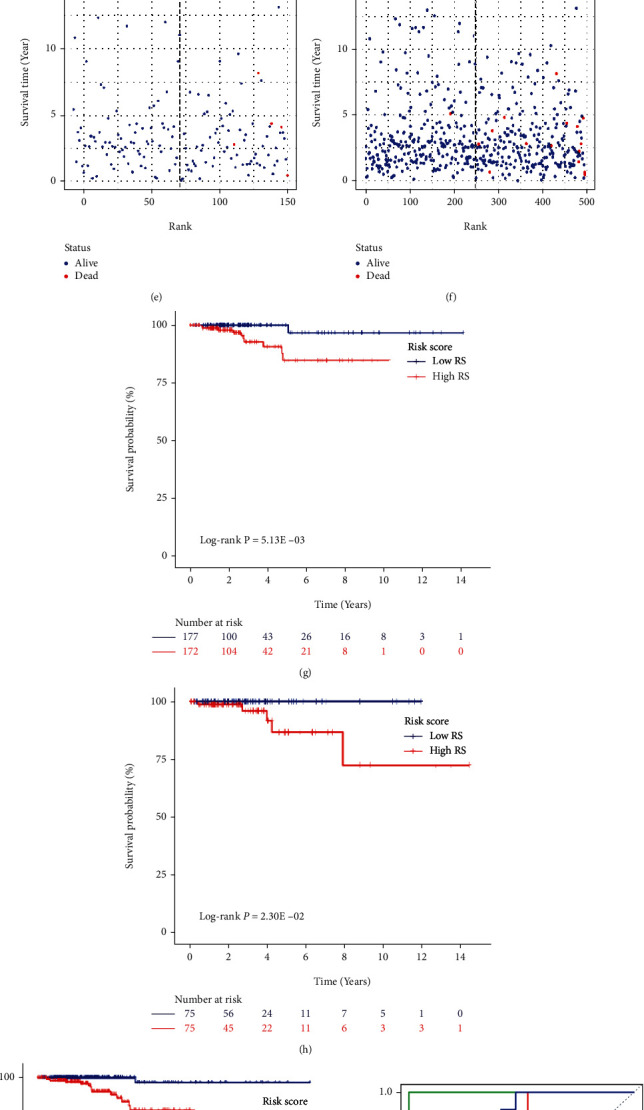
Validation of the four-gene Signature. (a–f) Risk score distribution and survival overview in the training cohort, testing cohort, and entire cohort. The horizontal axis depicts the ranking of patients in the cohort in terms of the risk score from lowest to highest. (g–i) The KM survival curves of risk score in the training cohort, testing cohort, and entire cohort. Patients were divided into high-risk and low-risk groups based on the median risk score. (j–l) The ROC curve of the risk score in the training cohort, testing cohort, and entire cohort.

The four-gene risk signature could effectively stratify patients into a high-risk group and a low-risk group with the median risk score. Thus, we could intuitively conclude that the patients who had died were basically the patients with higher ranking (higher risk score) (Figures 4(a)–4(f)). Consistent with this, significant differences of OS between the high-risk and low-risk groups in the training cohort (*P* = 5.13 × 10^−3^), testing cohort (*P* = 2.30 × 10^−2^), and the entire cohort (*P* = 2.56 × 10^−4^) were depicted by constructing KM curves (Figures 4(g)–4(i)). Moreover, the AUCs of the risk score corresponding to 1-year, 3-year, and 5-year survival were 1, 0.847, and 0.832 in the training cohort, 1, 0.836, and 0.947 in the testing cohort, and 0.855, 0.829, and 0.841 in the entire cohort, respectively (Figures 4(j)–4(l)). These results indicated that the four-gene risk signature exhibited useful value in accurately predicting the prognosis of PTC patients.

### 3.4. Building and Validating a Predictive Nomogram

**Figure 5:**
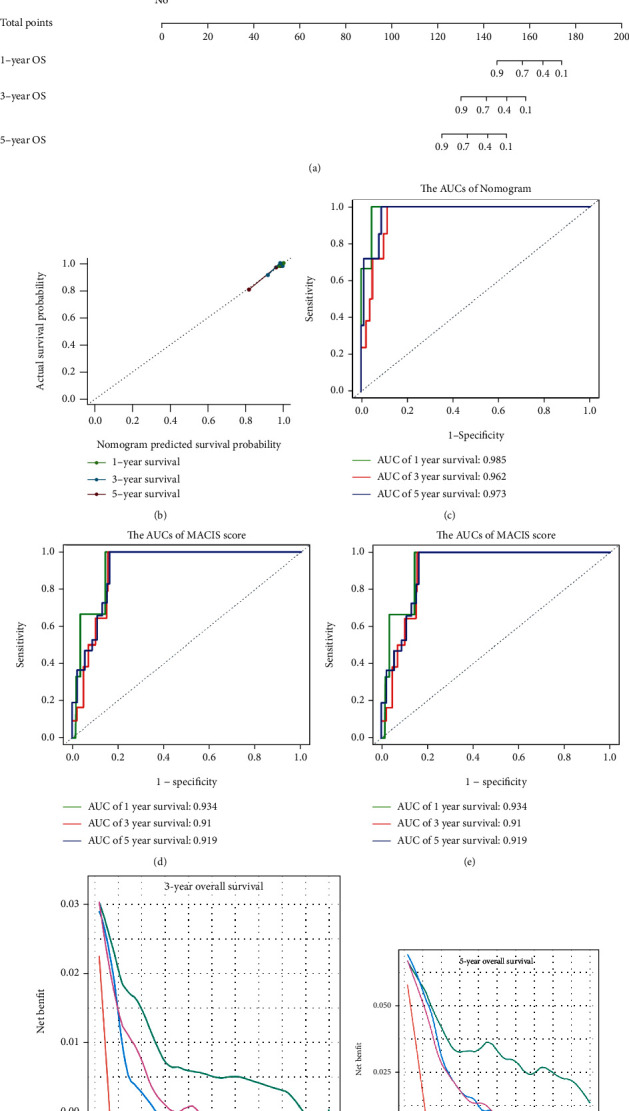
Construction of a nomogram for prediction of OS. (a) Nomogram was developed based on the risk scores and different clinical features. (b) Calibration plots were constructed to evaluate the predictive performance of OS. (c–e) The ROC curves of the AGES score, MACIS score, and the nomogram in the entire cohort. (f, g) The decision curves of AGES score, MACIS score, and the nomogram in the entire cohort.

Thereafter, in the entire cohort, univariate and multivariate Cox regression analyses were performed on the risk score and the clinical characteristics (Table 2). The risk score, age, AJCC stage, tumor size, extrathyroidal extension, and history of neoadjuvant treatment were selected to establish a nomogram (Figure 5(a)). The *C*-index, AIC, and BIC of this nomogram were 0.970, 85.743, and 86.451, respectively (Table 3). Moreover, a calibration curve revealed that the nomogram was excellent at predicting OS (Figure 5(b)). Moreover, the AUCs of the nomogram corresponding to 1-year, 3-year, and 5-year OS were 0.985, 0.962, and 0.973, respectively (Figure 5(c)). They were found to be significantly better than the traditional (Age, Grade, Extrathyroidal extension, Size) AGES score and (Metastases, Age, Completeness of resection, Invasion, Size) MACIS score (Figures 5(d) and 5(e)). Moreover, the decision curves of 3-year OS and 5-year OS of these three models indicated that in terms of predicting OS of PTC patients, the net benefit [17] of the nomogram model was substantially higher than that of the traditional AGES and MACIS score (Figures 5(f) and 5(g)). Taken together, these results revealed that this nomogram performed well in predicting the prognosis of PTC patients.

### 3.5. Immune Analysis

**Figure 6:**
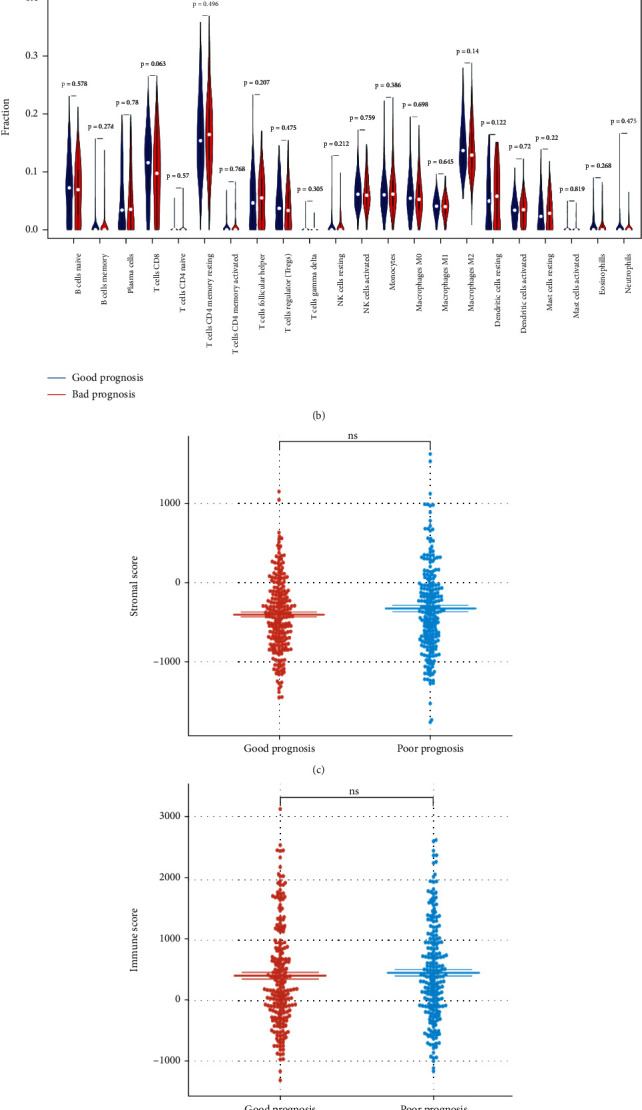
Immune analysis. (a, b) Immune infiltration of 22 different types of immune cells. (c–e) The stromal scores, immune scores, and estimate scores between the good-prognosis group and poor-prognosis group.

The nomogram could effectively stratify patients into a good-prognosis group and a poor-prognosis group based on the median total point. Surprisingly, immune analysis demonstrated that there was no significant difference in the abundance of 22 types of immune cells between the good-prognosis and poor-prognosis groups (Figures 6(a) and 6(b)). Consistent with this, the stroma scores, immune scores, and ESTIMATE scores were observed to be not significantly higher in the poor-prognosis group compared with the good-prognosis group (Figures 6(c)–6(e)), thus suggesting that the proportion of the stromal cells to immune cells in these two groups may be similar, but there was no difference in the tumor purity. These results implied that immune infiltration and immune microenvironment had minimal effect on the OS of PTC patients.

### 3.6. Gene Set Enrichment Analysis (GSEA) and Gene Set Variation Analysis (GSVA)

**Figure 7:**
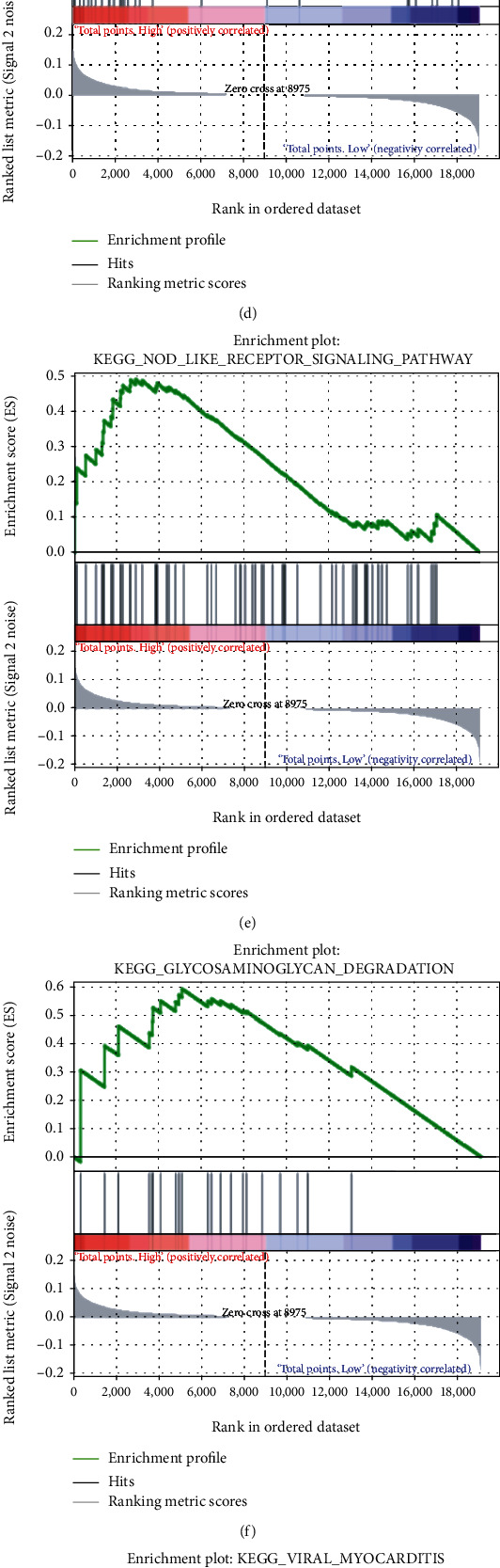
(a–h) Gene Set Enrichment Analysis (GSEA) and (i) Gene Set Variation Analysis (GSVA) in the entire cohort.

Since the immune infiltration analysis did not yield significant results, GSEA and GSVA were performed to further explore the potential differences in the molecular mechanisms between the good-prognosis and poor-prognosis groups. As shown in Figures 7(a)–7(h), the “ribosome,” “intestinal immune network for IgA production,” “systemic lupus erythematosus,” “asthma,” “nod like receptor signaling,” “glycosaminoglycan degradation,” “viral myocarditis,” and “cell adhesion molecules cams” pathways were found to be significantly enriched in the poor-prognosis group. Moreover, GSVA demonstrated that “peroxisome,” “myc targets V2,” “uv response up,” and “cholesterol homeostasis” were the hallmark pathways modulated in the poor-prognosis group (*t* value > 2) (Figure 7(i)). This implied that these pathways or the genes regulating them could play an important role in promoting the progression of PTC.

### 3.7. Construction of a ceRNA Regulatory Network

**Figure 8:**
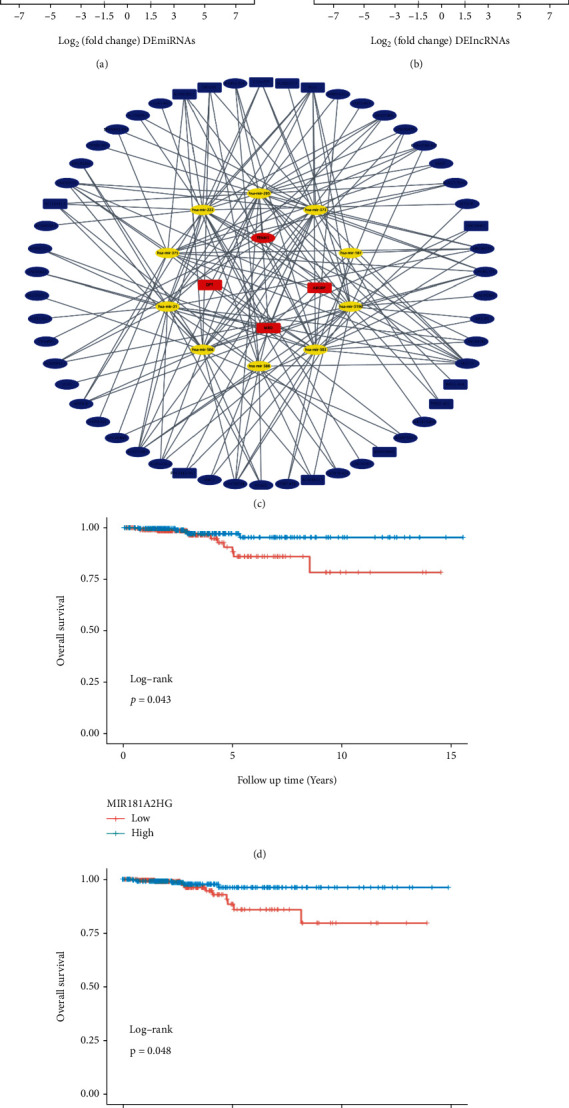
Construction of a ceRNA regulatory network. (a, b) DElncRNAs and DEmiRNAs between the normal tissues and PTC tissues in the TCGA-THCA dataset. (c) CeRNA network in TCGA-THCA dataset. Blue, yellow, and red represent DElncRNAs, DEmiRNAs, and DEmRNAs, respectively. (d, e) The KM curves of MIR181A2HG and hsa-mir-375.

95 DEmiRNAs (Table S5) and 839 DElncRNAs (Table S6) between the normal and PTC samples in the TCGA-THCA dataset were screened (Figures 8(a) and 8(b)). A total of 54 DElncRNAs and 10 DEmiRNAs were paired into 151 DElncRNA-DEmiRNA interactions, whereas 10 DEmiRNAs and 4 DEmRNAs were matched to form 15 pairs of DEmiRNA-DEmRNA interactions (Table S7). Consequently, the lncRNA-miRNA-mRNA ceRNA regulatory network, which contained 68 distinct nodes and 166 edges was constructed (Figure 8(c)). Among these, DElncRNAs and DEmiRNAs, KM curves showed that one lncRNA (MIR181A2HG) and one miRNA (hsa−mir−375) could serve as potential protective biomarkers in PTC patients (Figures 8(d) and 8(e)).

### 3.8. Validation of the Expression of the Four Genes in PTC Tissues

**Figure 9:**
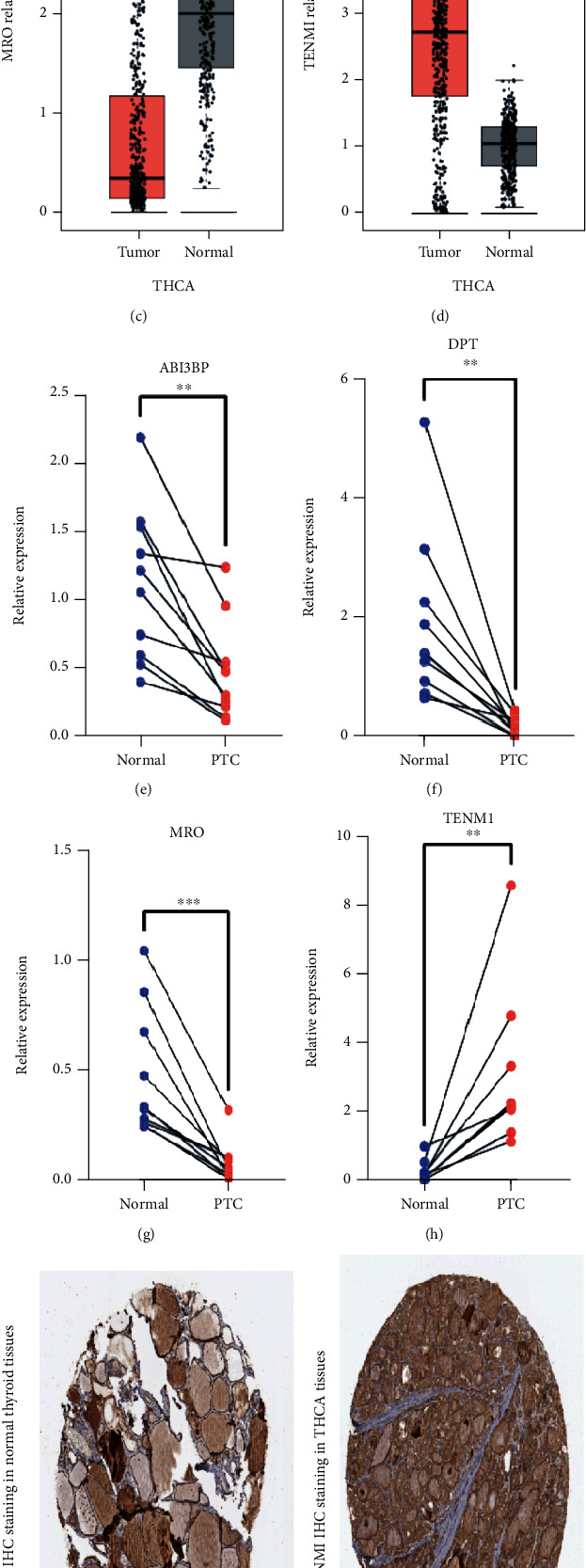
Expression of the four genes in the normal and PTC samples. (a–d) The mRNA expression of *ABI3BP*, *DPT*, *MRO*, and *TENM1* in the normal and PTC samples. The data was obtained from the GEPIA database. (e–h) The expression of selected four genes in the normal and PTC samples was analyzed using quantitative RT-PCR (*n* = 10). (i–k) Representative IHC images of TENM1 in the normal thyroid tissues and in PTC tissues. The data was retrieved from HPA database.

Among the four genes in four-gene risk signature, the levels of *ABI3BP*, *DPT*, and *MRO* were downregulated, whereas that of *TENM1* was upregulated in PTC samples (Figures 9(a)–9(d)). Thereafter, we used quantitative RT-PCR to verify the expression of these four genes in 10 pairs of PTC tissues (Figures 9(e)–9(h)). Furthermore, immunohistochemical staining data of TENM1 in the normal and tumor tissues were obtained by searching the HPA database, and higher protein expression of TENM1 was observed in analyzed tumor tissues (Figures 9(i)–9(k)).

## 4. Discussion

It has been established that even when the patients receive the standardized treatment, about 5%-23% of PTC patients display a poor prognosis [18]. Therefore, prediction of the prognosis of PTC patients can not only promote the active implementation of individualized treatment but also aid to avoid the various negative effects associated with excessive medical treatment. A number of prognostic markers obtained from gene expression profiles can accurately predict the prognosis of a single patient at the molecular level and can be complementary to the traditional clinical staging prediction system such as TNM staging [19].

In the present study, a novel four-gene risk signature comprising *ABI3BP*, *DPT*, *MRO*, and *TENM1* to predict the OS of PTC was identified. The efficacy of this signature was validated in the study cohort. Among these four genes, the levels of *ABI3BP*, *DPT*, and *MRO* were downregulated whereas that of *TENM1* was upregulated in PTC. *ABI3BP* is an ArgBP/E3B1/Avi2/NESH family protein, which can participate in the negative regulation of the cell movement and metastasis through its influence on membrane folding and layer formation [20, 21]. It has been proven to be an src-homologous 3(SH3) adapter molecule and can exhibit a tumor-suppressive effect in thyroid cancer [22, 23]. *ABI3BP*, which is reexpressed in the thyroid cells, has been reported to trigger cellular senescence through affecting the p21 pathway, resulting in a reduction in transformation activity, cell growth, viability, migration, invasion, and tumor growth in nude mice [22]. Moreover, the loss of *ABI3BP* expression may be functionally involved in the pathogenesis of several types of cancer such as gallbladder cancer [24] and esophageal cancer [25]. *DPT* is a tyrosine-rich noncollagenous extracellular matrix component, and the depletion of *DPT* has been associated with hyperproliferation of scars, skin fibrosis, systemic sclerosis as well as some cancers [26]. *DPT* has been reported to regulate cell proliferation and invasiveness of a variety of tumors like endometrial cancer [27], prostate cancer [28], hepatocellular carcinoma [29], and oral cancer [30]. Moreover, low *DPT* expression in PTC has been related to higher T classification. *DPT* can regulate *CDK4*, *CDK6*, and *p21* through modulating MEK-ERK-MYC signaling to inhibit PTC proliferation [31]. The expression of *TENM1* as a cell signal sensor in neurons has been positively correlated with the growth and invasion of PTC. *TENM1* has been identified as the direct functional target of miR-486 in PTC cells. The restoration of miR-486 can significantly inhibit the growth of PTC *in vivo* [32, 33]. Similar to the previous two genes, *TENM1* can also play an important role in different types of cancer especially in the brain tumors such as prolactin pituitary tumor [34] and glioblastoma [35]. *MRO* belongs to a novel gene family, named “maestro heat-like repeat family (MROH)” [36]. Expression of *MRO* in lean-type polycystic ovarian syndrome has been found to be increased [37]. Nevertheless, *MRO* has been poorly studied in the tumors, and the role of *MRO* in thyroid cancer has not been previously reported. We speculate that *MRO*, as a sex-determining gene affecting the prognosis of PTC, might be related to the sex differences between PTC patients, but this observation needs further analysis.

The 8th edition of the AJCC/TNM staging system (TNM-8th) was released in 2017. A number of studies have proved that it was more appropriate for the prediction of the survival and recurrence than TNM-7th [7, 38]. However, studies also have shown that approximately 30%-40% [39, 40] of patients were downstaged upon reclassification. In addition, TNM-8th oversimplifies the survival deterioration that primarily occurs with increasing age at the time of diagnosis and underestimates the prognosis for the younger patients, particularly those aged 45–55 years [9]. What is more, the molecular profile, which is important for the precision medicine, has not been contained in TNM-8th. Consequently, as knowledge of cancer biology evolves, innovative diagnostic tools and treatment modalities need to be developed and improved. Novel nomograms for predicting the survival of thyroid cancer patients have been previously formulated in several studies. For instance, Pathak and colleagues developed nomograms to predict the likelihood of the relapse and death from thyroid cancer in an individual patient (*C*-indices were 0.92 and 0.76, respectively) based on patients' characteristics but without identification of any specific gene signature [41]. A nomogram that first included a gene signature to predict 1-year, 3-year, and 5-year DFS of DTC patients was established (*C*‐index = 0.801) by Pan Ruchong [42]. However, five-gene signature appears to cause significant expenditure in the healthcare compared to the four-gene signature. In addition, to the best of our knowledge, our study is the first study to confirm that the developed nomogram performed better than the traditional AGES score and MACIS score for prediction of OS. Besides, our study demonstrated that BRAF mutations had minimal effect on OS of PTC. Consistent with this, unlike previous studies suggesting that *BRAF* mutations can imply poor prognosis [43], some recent studies have also indicated that *BRAF* mutations cannot be used as an independent prognostic and predictive factor in PTC [44]. Moreover, Wang et al. found that compared to male PTC patients, *BRAF* mutations cannot be considered as robust independent risk factor for female patients [45]. In general, the nomogram constructed in this study exhibited better predictive efficacy for the OS of PTC patients and clinical applicability.

To understand the potential mechanisms affecting the prognosis of patients with PTC, immune analysis, GSEA, and GSVA were performed between good-prognosis and poor-prognosis groups. Surprisingly, in contrast to some other prior studies [46], immune infiltration and immune microenvironment displayed little effect on the OS of PTC patients. These results can be explained, in part, by the fact that the four key genes we identified in this study are not directly involved in the process of tumor immunity. Therefore, no significant biological difference in immune infiltration was observed when PTC samples were divided into good and poor prognosis groups. The “cell adhesion molecules cams” [47], “Myc” pathways [48], and glycosaminoglycans (GAGs) [49], which can play an essential role in the tumor pathogenesis and distant metastasis were found to be enriched in poor-prognosis groups. The effects of intestinal immunity caused by intestinal flora on the various cancers are currently being elucidated, including gastrointestinal tumors, liver cancer [50], and breast cancer [51]. In addition, intestinal immunity can also affect the thyroid function [52]. However, the effect of intestinal immunity on PTC remains to be further studied. Moreover, a number of studies have reported that systemic lupus erythematosus (SLE) and asthma were associated with an increased risk of overall cancers including non-Hodgkin's lymphoma, Hodgkin's lymphoma, leukemia, multiple myeloma, and thyroid cancer. Therefore, the increased expression of genes associated with lupus and asthma in the poor-prognosis group is understandable. What is more, the key molecules were presented in the ceRNA network including MIR181A2HG and hsa-mir-375, which need to be studied in detail in PTC.

Although this study revealed numerous important findings, but there are several limitations associated with it. First, selection bias and confounding bias were inevitable due to the retrospective design of this study. Second, the clinical characteristics were mainly derived from TCGA database, and thus, caution should be exercised when expanding our results to patients of other ethnicities. Besides, in future studies, the nomogram should be validated in different external datasets. Finally, additional *in vitro* and *in vivo* functional experiments need to be performed to further elucidate the detailed molecular mechanisms affecting the prognosis of PTC.

## 5. Conclusion

To conclude, our study established a four-gene risk signature and developed a novel prognostic nomogram in combination with prognosis-related clinical characteristics to predict the OS of PTC. The four DEGs were found closely related to the prognosis of PTC and thus can act as potential therapeutic targets. These results might be beneficial for individualized treatment and medical decision-making during the management of PTC patients.

## Figures and Tables

**Table 1:** The various clinical characteristics in the training, testing, and entire cohort.

Clinical characteristics	Training cohort (344)	Testing cohort (150)	Entire cohort (494)
Histological type			
PTC	269	118	387
FVPTC	71	29	100
Unknown	4	3	7
Age			
<55	232	98	330
≥55	112	52	164
Gender			
Male	92	42	134
Female	252	108	360
Tumor size			
≤1 cm	26	11	37
>1 cm	291	128	419
Unknown	27	11	38
Neoadjuvant treatment			
Yes	9	5	14
No	335	145	480
Focality			
Unifocal	182	79	261
Multifocal	155	68	223
Unknown	7	3	10
Site			
Unilateral	267	115	382
Bilateral	60	25	85
Isthmus	15	7	22
Unknown	2	3	5
Extrathyroidal extension			
None	227	97	324
Minimal	92	40	132
Gross	13	6	19
Unknown	12	7	19
AJCC stage			
I	195	84	279
II	37	15	52
III	75	35	110
IV	37	16	53
M			
M0	195	86	281
M1	7	1	8
MX	142	63	205
N			
N0	155	71	226
N1	155	65	220
NX	34	14	48
T			
T1	96	43	139
T2	116	46	162
T3	116	53	169
T4	14	8	22
TX	2	0	2
Radiation			
Yes	212	90	302
No	121	55	176
Unknown	11	5	16
BRAF mutation			
Yes	193	82	275
No	135	62	197
Unknown	16	6	22
Overall survival (year)			
Mean ± SD	3.208 ± 1.205	3.58 ± 0.24	3.323 ± 0.121

**Table 2:** Cox regression of the clinical characteristic and the risk score.

Variables	Univariate analysis		Multivariate analysis	
	HR (95% CI)	*P*	HR (95% CI)	*P*
Risk score	2.718 (1.888, 3.914)	**<0.001**	2.37 (1.40, 4.00)	**0.001**
Age	1.119 (1.077, 1.163)	**<0.001**	1.10 (1.050, 1.21)	**0.045**
Race (white)	1.255 (0.284, 5.536)	0.765	—	—
Gender (male vs. female)	1.921 (0.692, 5.324)	0.209	—	—
Neoadjuvant treatment (yes)	23.55 (4.967, 111.7)	**<0.001**	22.59 (2.40-45.22)	**0.006**
Histology (PTC vs. FVPTC)	3.162 (0.421, 23.953)	0.266	—	—
Site (unilateral vs. bilateral)	0.847 (0.186, 3.866)	0.831	—	—
Site (isthmus vs. bilateral)	0.970 (0.086, 10.929)	0.981	—	—
Focality (unifocal vs. multifocal)	3.918 (0.884, 17.36)	0.072	—	—
Tumor size	1.362 (1.048, 1.769)	**0.021**	1.25 (1.05-1.57)	**0.034**
Extrathyroidal extension (minimal vs. gross)	0.110 (0.029, 0.413)	**0.001**		**0.012**
Extrathyroidal extension (none vs. gross)	0.100 (0.031, 0.316)	**<0.001**		**0.003**
M (M1 vs. M0)	4.852 (1.042, 22.681)	**0.045**	2 (0.31, 12.96)	0.468
M (MX vs. M0)	4.852 (1.049, 22.683)	0.529	—	—
N (N1 vs. N0)	1.443 (0.472, 4.425)	0.522	—	—
N (NX vs. N0)	2.45 (0.58, 10.27)	0.221	—	—
T (T2 vs. T1)	1.04 (0.17, 6.23)	0.968	0.72 (0.11, 4.74)	0.736
T (T3 vs. T1)	1.57 (0.3, 8.15)	0.591	0.53 (0.08, 3.29)	0.493
T (T4 vs. T1)	11.73 (2.34, 58.78)	**0.003**	2.66 (0.36, 19.55)	0.335
T (TX vs. T1)	0 (0, Inf)	0.998	0 (0, Inf)	0.999
AJCC stage (II vs. I)	5.26 (0.74, 37.61)	0.098	3.39 (0.36, 31.58)	0.284
AJCC stage (III vs. I)	9.53 (1.98, 45.96)	**0.005**	6.45 (1.08, 38.4)	**0.041**
AJCC stage (IV vs. I)	19.27 (3.7, 100.35)	**<0.001**	6.29 (3.75, 12.92)	**0.034**
Radiation (yes vs. no)	1.29 (0.4, 4.12)	0.666	—	—
BRAF mutation (yes vs. no)	0.618 (0.223, 1.714)	0.355	—	—

**Table 3:** The *C*-index, AIC, and BIC of the different models.

Models	*C*-index	AIC	BIC
Nomogram	0.970	85.743	86.451
MACIS score	0.928	112.410	113.118
AGES score	0.923	116.589	117.297
Risk score	0.847	119.816	120.524
AJCC stage	0.793	137.890	138.598
Age	0.832	123.993	124.701
Tumor size	0.65	152.771	153.479
Extrathyroidal extension	0.712	147.590	148.298
History of neoadjuvant treatment	0.548	149.542	150.250

## Data Availability

The datasets used and/or analyzed during the current study are available from the corresponding author on reasonable request.

## Supplementary Materials

Supplementary 1 Figure 1: value distribution of the selected samples in the three GEO datasets. Figure 2: identification of DGEs. (A-D) Volcano maps in the three GEO datasets and TCGA-THCA dataset. (E) The intersection of the four datasets contained 176 DEGs.

Supplementary 2 Table S1: details of the GEO and TCGA datasets used in this study. Table S2: samples in HPA database. Table S3: the sequences of primers. Table S4: univariate Cox regression of the 176 genes in the training cohort. Table S5: 96 DEmiRNAs between PTC and normal thyroid tissues. Table S6: 839 DEIncRNAs between PTC and normal thyroid tissues. Table S7: the IncRNAs, mARNAs, and miRNAs in the ceRNA network.
