# Supplementary figures and images for: A Potential Four-Gene Signature and Nomogram for Predicting the Overall Survival of Papillary Thyroid Cancer
Source: Dis Markers. 2022 Aug 30;2022:8735551. doi: 10.1155/2022/8735551 (PMC9526076; doi:10.1155/2022/8735551)

**GSE3678**

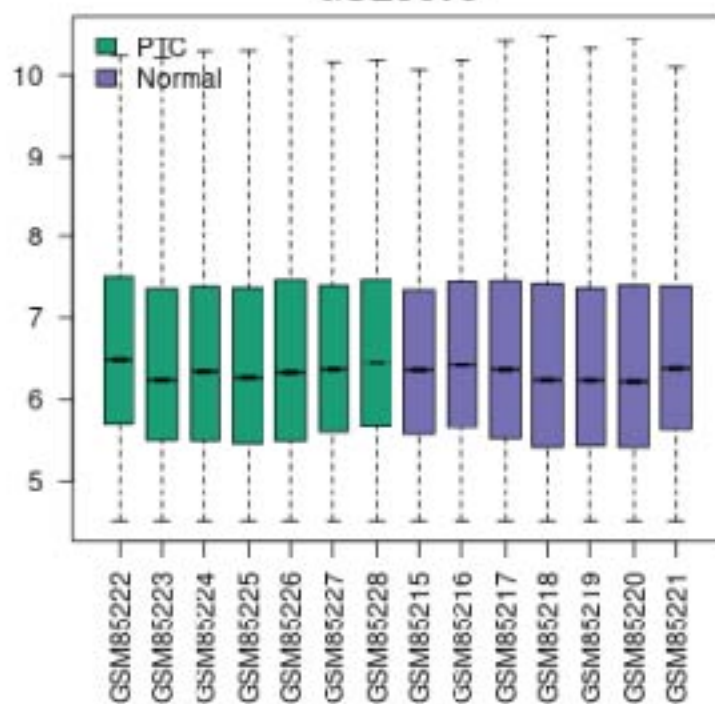

B

**GSE60542, selected samples**

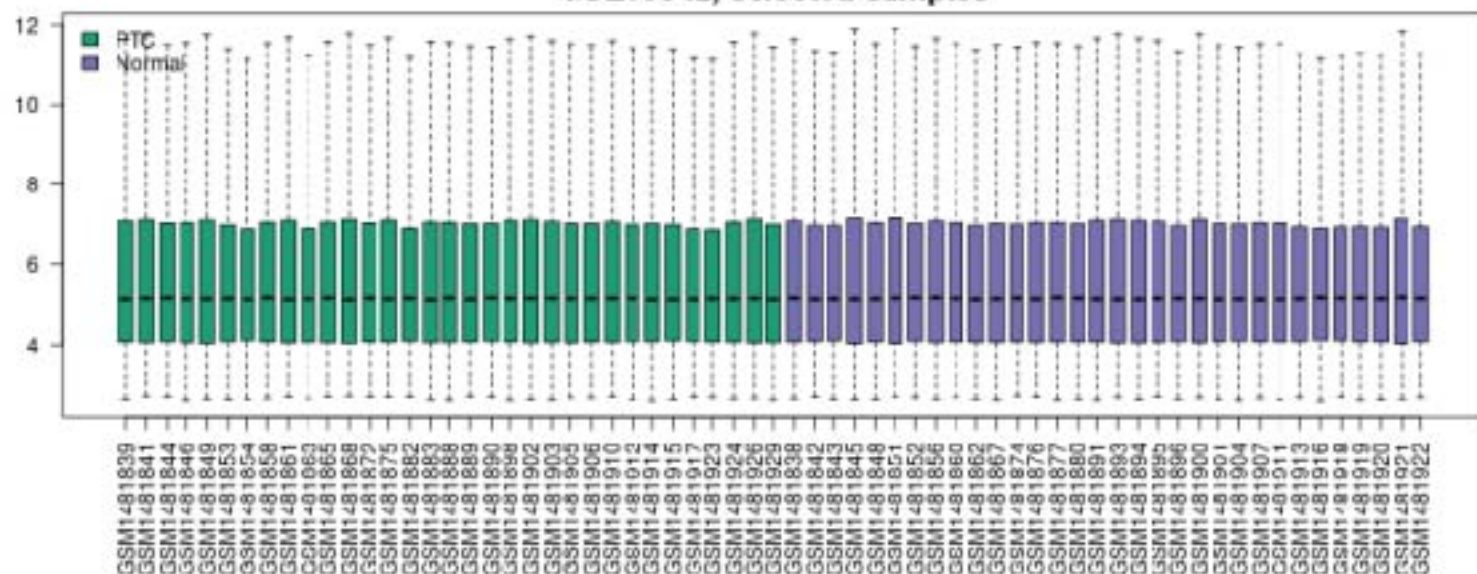

C

GSE33630, selected samples

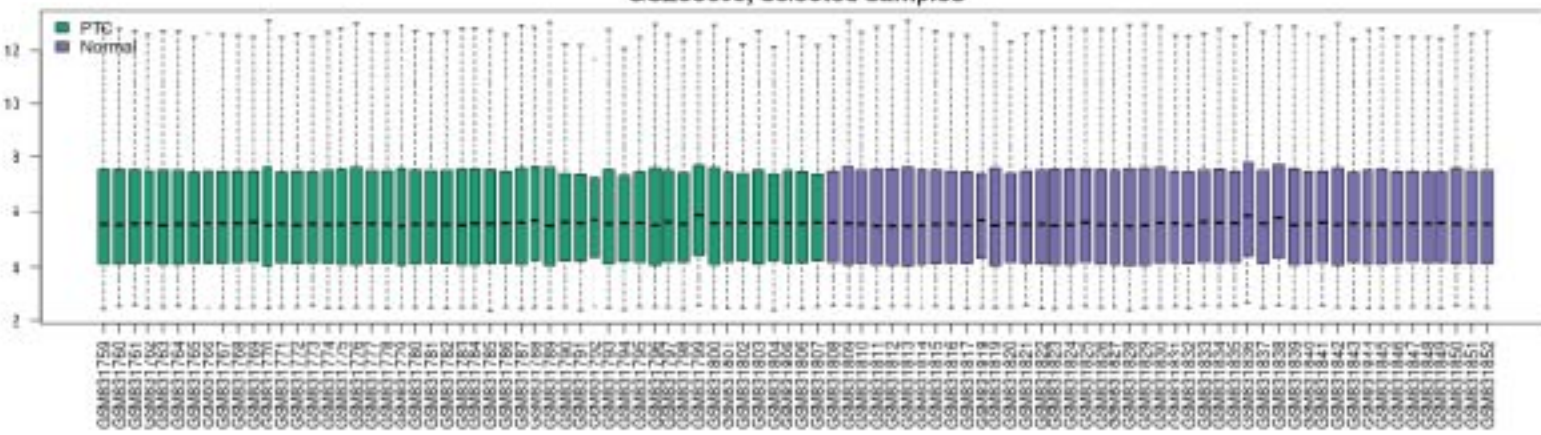

Supplement: Supplementary 1 — Figure 1: value distribution of the selected samples in the three GEO datasets. Figure 2: identification of DGEs. (A-D) Volcano maps in the three GEO datasets and TCGA-THCA dataset. (E) The intersection of the four datasets contained 176 DEGs. [file 8735551.f1.zip › supplementary Figure 1.pdf]

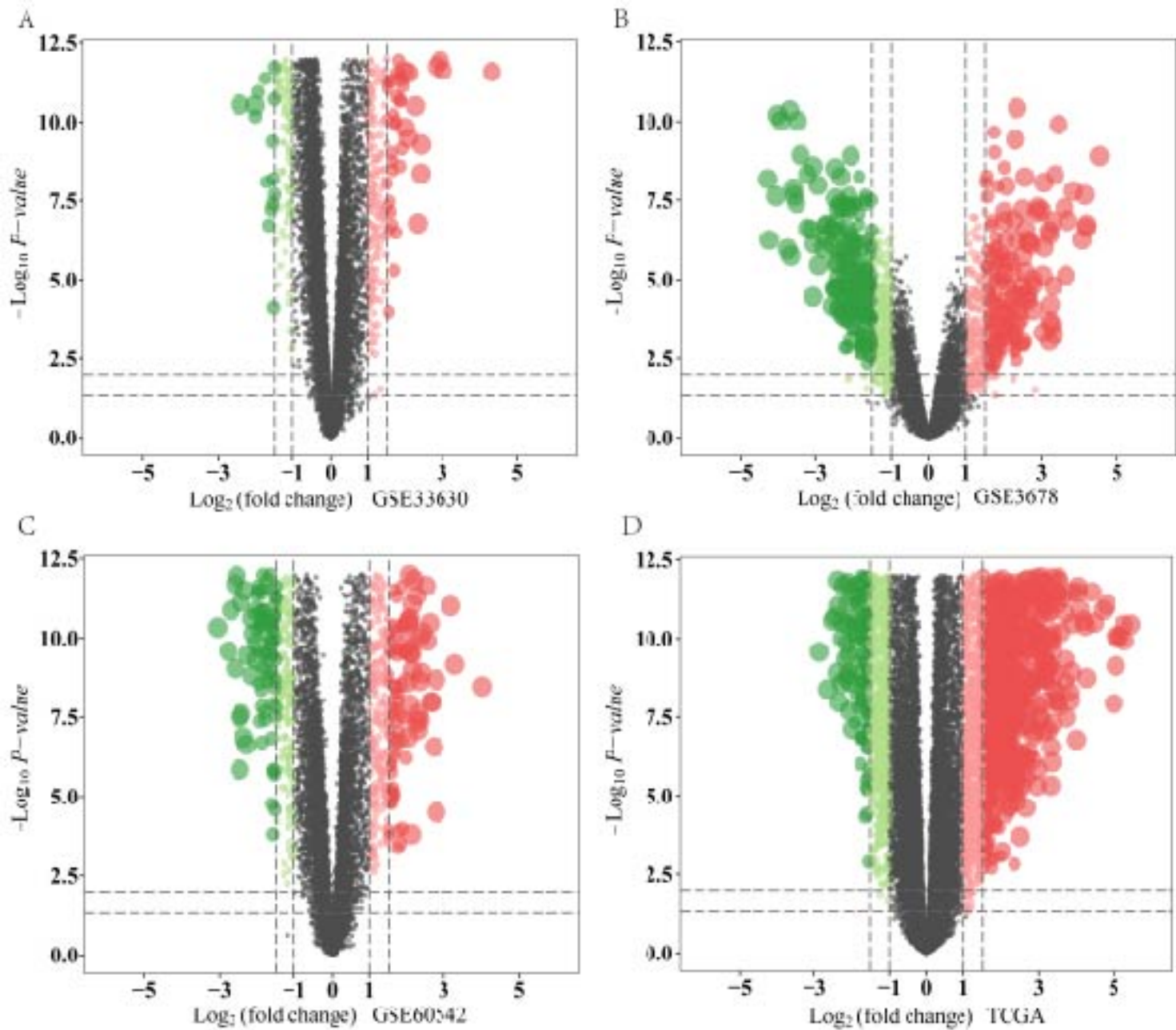

**E**

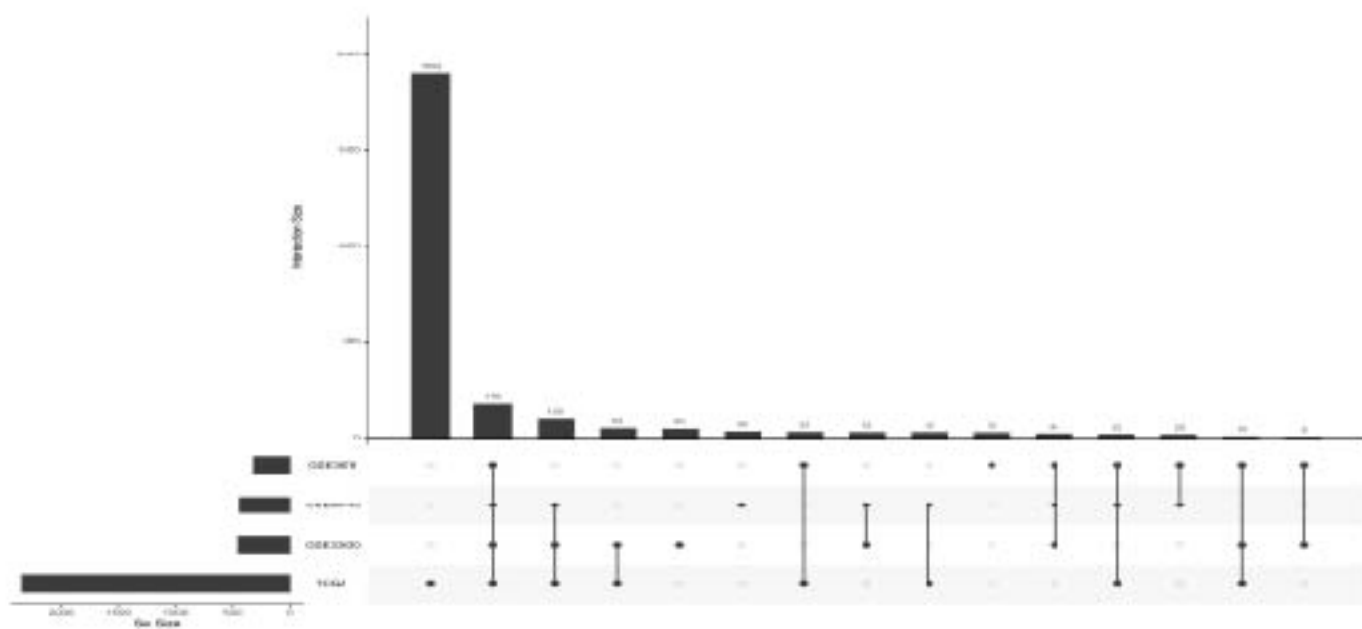

Supplement: Supplementary 1 — Figure 1: value distribution of the selected samples in the three GEO datasets. Figure 2: identification of DGEs. (A-D) Volcano maps in the three GEO datasets and TCGA-THCA dataset. (E) The intersection of the four datasets contained 176 DEGs. [file 8735551.f1.zip › supplementary figure 2.pdf]
