# Supplementary material for: A Potential Four-Gene Signature and Nomogram for Predicting the Overall Survival of Papillary Thyroid Cancer
Source: Dis Markers. 2022 Aug 30;2022:8735551. doi: 10.1155/2022/8735551 (PMC9526076; doi:10.1155/2022/8735551)
Supplement: Supplementary 2 — Table S1: details of the GEO and TCGA datasets used in this study. Table S2: samples in HPA database. Table S3: the sequences of primers. Table S4: univariate Cox regression of the 176 genes in the training cohort. Table S5: 96 DEmiRNAs between PTC and normal thyroid tissues. Table S6: 839 DEIncRNAs between PTC and normal thyroid tissues. Table S7: the IncRNAs, mARNAs, and miRNAs in the ceRNA network. [file 8735551.f2.zip › Table S1 (1).docx]

Table S1 Details of the GEO and TCGA datasets used in this study.

| Datasets | Platform | Sample size (tumor/normal) | Application |
| --- | --- | --- | --- |
| GSE33630 | [HG-U133_Plus_2] Affymetrix Human Genome U133 Plus 2.0 Array | 94(49/45) | Identification of DEGs |
| GSE3678 | [HG-U133_Plus_2] Affymetrix Human Genome U133 Plus 2.0 Array | 14(7/7) | Identification of DEGs |
| GSE60542 | [HG-U133_Plus_2] Affymetrix Human Genome U133 Plus 2.0 Array | 61(32/29) | Identification of DEGs |
| TCGA-THCA | Illumina RNA Sequencing | 568(510/58) | Identification of DEGs, model construction and validation |
