# Supplementary material for: A Potential Four-Gene Signature and Nomogram for Predicting the Overall Survival of Papillary Thyroid Cancer
Source: Dis Markers. 2022 Aug 30;2022:8735551. doi: 10.1155/2022/8735551 (PMC9526076; doi:10.1155/2022/8735551)
Supplement: Supplementary 2 — Table S1: details of the GEO and TCGA datasets used in this study. Table S2: samples in HPA database. Table S3: the sequences of primers. Table S4: univariate Cox regression of the 176 genes in the training cohort. Table S5: 96 DEmiRNAs between PTC and normal thyroid tissues. Table S6: 839 DEIncRNAs between PTC and normal thyroid tissues. Table S7: the IncRNAs, mARNAs, and miRNAs in the ceRNA network. [file 8735551.f2.zip › Table S2.docx]

| ID | Sex | Age | Tissue | Immunohistochemical staining |
| --- | --- | --- | --- | --- |
| 2146 | Female | 22 | Normal thyroid gland | Medium |
| 1501 | Female | 75 | Normal thyroid gland | Medium |
| 2072 | Male | 61 | Normal thyroid gland | Medium |
| 688 | Male | 20 | PTC tissue | High |
| 515 | Male | 33 | PTC tissue | High |
| 1014 | Female | 35 | PTC tissue | High |
| 2189 | Female | 91 | PTC tissue | Medium |

Table S2. Samples in HPA database.
