# Supplementary material for: A Potential Four-Gene Signature and Nomogram for Predicting the Overall Survival of Papillary Thyroid Cancer
Source: Dis Markers. 2022 Aug 30;2022:8735551. doi: 10.1155/2022/8735551 (PMC9526076; doi:10.1155/2022/8735551)
Supplement: Supplementary 2 — Table S1: details of the GEO and TCGA datasets used in this study. Table S2: samples in HPA database. Table S3: the sequences of primers. Table S4: univariate Cox regression of the 176 genes in the training cohort. Table S5: 96 DEmiRNAs between PTC and normal thyroid tissues. Table S6: 839 DEIncRNAs between PTC and normal thyroid tissues. Table S7: the IncRNAs, mARNAs, and miRNAs in the ceRNA network. [file 8735551.f2.zip › Table S3 (1).pdf]

Table S3. The sequences of the primers

Primer	Sequence (5’ to 3’)	Number of bases
MRO-F	TAAAAAGCGTCACATGGCAATG	22
MRO-R	GCAGGTCGAGGACAATTTTCTT	22
ABI3BP-F	CAAATGCAACATGCTCTCCAGT	22
ABI3BP-R	TTGGCCTTTTACCTTTTGGCA	21
DPT-F	TGACAGACAATGGAACTACGC	21
DPT-R	TGCTGTAGCGACAACAGTAAAA	22
TENM1-F	ACTGGAGAAGTTGACATTGGTG	22
TENM1-R	GTGGATAGTAATCTGGAAACGCC	23
