# Supplementary material for: A Potential Four-Gene Signature and Nomogram for Predicting the Overall Survival of Papillary Thyroid Cancer
Source: Dis Markers. 2022 Aug 30;2022:8735551. doi: 10.1155/2022/8735551 (PMC9526076; doi:10.1155/2022/8735551)
Supplement: Supplementary 2 — Table S1: details of the GEO and TCGA datasets used in this study. Table S2: samples in HPA database. Table S3: the sequences of primers. Table S4: univariate Cox regression of the 176 genes in the training cohort. Table S5: 96 DEmiRNAs between PTC and normal thyroid tissues. Table S6: 839 DEIncRNAs between PTC and normal thyroid tissues. Table S7: the IncRNAs, mARNAs, and miRNAs in the ceRNA network. [file 8735551.f2.zip › Table S4 (1).pdf]

Table S4. Univariate cox regression of the 176 genes in the training cohort.

Gene	HR	P
**DPT**	**0.810723712**	**0.000231088**
**ANK2**	**0.543507697**	**0.000239084**
**PAPSS2**	**0.383742375**	**0.000347**
**MRO**	**0.655850716**	**0.000447167**
**AOX1**	**0.786535498**	**0.000483401**
**GHR**	**0.404297319**	**0.000952555**
**CWH43**	**0.812824954**	**0.001284228**
**ABI3BP**	**0.62932062**	**0.002107004**
**CCL21**	**0.866689267**	**0.00272971**
**PID1**	**0.719336586**	**0.003429462**
**SLC4A4**	**0.718202559**	**0.00426557**
**RASSF9**	**0.724837736**	**0.004994975**
**CITED1**	**1.621063109**	**0.007120717**
**RASSF6**	**0.634675318**	**0.008055402**
**NCAM1**	**0.730439269**	**0.008736826**
**RNF150**	**0.579781153**	**0.008759491**
**TENM1**	**1.541907099**	**0.018653458**
**RYR2**	**0.684620374**	**0.018988846**
**DLG2**	**0.609771099**	**0.019885771**
**QPCT**	**1.961785065**	**0.020838637**
**GALE**	**2.457506556**	**0.02348752**
**TPO**	**0.831922415**	**0.025883467**
**IP6K3**	**0.755547593**	**0.026685046**
**TIMP1**	**1.594061419**	**0.027414043**
**ZMAT4**	**0.898009645**	**0.027900822**
**SCARA5**	**0.897099444**	**0.02828766**
**STK32A**	**1.969715724**	**0.029926649**
**RXRG**	**1.859855769**	**0.030545756**
**DPP6**	**0.849059176**	**0.033593659**
**GOLT1A**	**1.839717769**	**0.034001283**
**CAMK2N1**	**1.676931407**	**0.035464096**
**TFCP2L1**	**0.711619482**	**0.03572262**
**SERTM1**	**0.903915017**	**0.039255435**
**MATN2**	**0.64836001**	**0.040623726**
**CTSH**	**1.64003329**	**0.047648007**
KIAA1324	0.751180004	0.052296279
WSCD2	0.857884587	0.055236207
COL13A1	1.510595126	0.056435491
GDF15	1.393130178	0.056985814
NOD1	1.610325802	0.05809079
ABCC3	1.659276064	0.063586687
DEPTOR	0.645836804	0.064702621
PKHD1L1	0.902387036	0.066058122
APOD	0.805817847	0.066537922
GALNT7	2.200292754	0.068863759
PROM1	0.871936132	0.069133416
CDH16	0.825226152	0.075362796
ARHGAP24	0.742592666	0.075756342
NTM	0.792629058	0.079654243

TNFRSF11B	0.765722196	0.080240325
PDLIM4	1.428759431	0.081458123
ARHGAP36	1.133309399	0.082989682
IPCEF1	0.709177371	0.08318854
HMGA2	1.429336832	0.08363355
CDH3	1.634250911	0.083719421
EPHB1	0.666606328	0.085249143
SORBS2	0.657681191	0.085455052
C4orf48	1.292796107	0.086711932
PPARGC1A	0.786267734	0.088516974
RELN	0.84980078	0.088671568
LRP1B	0.860836689	0.093824647
ELMO1	0.696327093	0.093941446
LPAR5	1.808933527	0.094800449
KIT	0.880967982	0.097290863
IGSF1	1.260151706	0.099933283
PROX1	0.761037154	0.104799641
DGKI	0.768518644	0.109181337
HGD	0.821416897	0.109462012
SMOC2	0.774821959	0.109756667
KLHDC8A	1.36045472	0.111064857
ADH1B	0.896130737	0.111180953
MAMLD1	1.427596878	0.115200338
LMOD1	0.706068886	0.117595754
GLT8D2	0.741247103	0.121196867
SDC4	1.546405916	0.124096069
TIAM1	1.414248095	0.124514684
PRSS2	1.13227789	0.126250026
FAXC	1.532137122	0.128365674
METTL7B	1.488323025	0.132645986
CCDC146	0.625154103	0.133170329
IGFBPL1	0.890685501	0.139462998
CST6	1.191383574	0.141391012
LIFR	0.734051218	0.142005713
PRR15	1.236766154	0.148882255
MRC2	1.425244541	0.151501236
MPPED2	0.832349279	0.159925926
GABRB2	1.218025439	0.164309475
UBE2QL1	1.418972511	0.165007322
AGR2	1.267777029	0.173097811
KCNJ2	1.267561241	0.175045813
SLC34A2	1.158486461	0.17680942
RUNX2	1.308136665	0.178667111
DTX4	1.336807764	0.18042517
ENTPD1	1.509993218	0.180683851
KLK10	1.14884403	0.18631363
SERPINA1	1.229939797	0.189063876
LGALS3	1.351384721	0.198774151
ZCCHC12	1.152764857	0.200244572
TFF3	0.88619559	0.201696621
ALOX15B	1.182956668	0.206995938

C11orf74	0.646970197	0.237269596
KCNN4	1.148303803	0.238841847
NGEF	1.188449057	0.240010655
PROS1	1.269799491	0.242106673
KCNQ3	1.217800464	0.246761569
LIPH	1.250860232	0.253265185
PLXNC1	1.254741843	0.253761723
PDE5A	1.283880032	0.257263396
SLC26A7	0.898646086	0.258309674
CFI	1.322507486	0.261375292
CLDN1	1.310866586	0.265967348
HEY2	1.424175707	0.274783224
SLIT1	1.119471056	0.289660483
SPX	0.870700982	0.2930075
GPM6A	0.858947973	0.293445358
SLC27A6	1.098921645	0.301737429
CITED2	0.768968434	0.3019425
AVPR1A	0.873231017	0.310542861
FRMD3	1.246065547	0.312534391
IL1RAP	1.224958983	0.31914618
SLC26A4	0.916533607	0.323237475
STXBP5L	0.930551739	0.32739291
CXCL14	1.076850244	0.327669865
SYTL5	1.117622117	0.332214161
BMP8A	0.885169111	0.336032662
LRRK2	1.171006228	0.353833218
DUSP5	1.156970061	0.368492293
DCSTAMP	1.062144286	0.375239215
TGFA	1.296210131	0.3767611
CHI3L1	1.081115294	0.381420001
KRT19	1.133014833	0.399190147
SDK1	1.119404099	0.404743681
FOXQ1	1.12885863	0.410477351
FAM20A	1.138181369	0.412579024
MET	1.18755361	0.423647936
IRS4	0.955859269	0.432126743
CRABP1	0.944663341	0.433014364
CLDN10	1.048812724	0.44660038
CYP1B1	1.096695557	0.461029784
LRP4	1.143453272	0.474562139
WDR72	0.890014276	0.488406111
ZFPM2	0.882542759	0.497845314
FN1	1.064157371	0.534784808
TMEM163	1.073064785	0.544215238
TCEAL2	0.943517894	0.562050665
CFD	0.882224008	0.567986032
ANGPTL1	0.901178069	0.574090562
THRSP	0.909971435	0.59392182
MUM1L1	0.943132819	0.59913334
TACSTD2	1.05322719	0.607372695
TMPRSS4	1.042788382	0.611818241

LAMP5	1.044977959	0.628566275
FAM84A	1.113257896	0.641756423
C19orf33	1.063325301	0.646860746
DPP4	1.06212217	0.650673108
LAMB3	1.052725749	0.663634145
CDKN2B	0.909082931	0.665319682
PCSK2	1.041090875	0.666951981
SCEL	1.064456485	0.68111634
GABBR2	1.062905482	0.681239002
ALDH1A3	0.949269959	0.706424311
NRCAM	1.117665137	0.707103067
CLCNKB	0.938856476	0.714803616
SLPI	0.966787721	0.714929882
AHNAK2	1.046035949	0.727312002
CEACAM6	0.980677164	0.74743981
HBB	0.962384647	0.816692327
NR2F1.AS1	0.968594107	0.824913921
NFE2L3	1.019660814	0.893913107
MMP16	1.014588546	0.912577863
PSD3	1.024106838	0.928904015
CSGALNACT1	0.979078749	0.935890395
ALOX5	0.99454567	0.958700182
CDH2	0.994054292	0.959444217
P4HA2	0.996274634	0.99223767
CTSC	1.001412857	0.994145363
