# Supplementary material for: A Potential Four-Gene Signature and Nomogram for Predicting the Overall Survival of Papillary Thyroid Cancer
Source: Dis Markers. 2022 Aug 30;2022:8735551. doi: 10.1155/2022/8735551 (PMC9526076; doi:10.1155/2022/8735551)
Supplement: Supplementary 2 — Table S1: details of the GEO and TCGA datasets used in this study. Table S2: samples in HPA database. Table S3: the sequences of primers. Table S4: univariate Cox regression of the 176 genes in the training cohort. Table S5: 96 DEmiRNAs between PTC and normal thyroid tissues. Table S6: 839 DEIncRNAs between PTC and normal thyroid tissues. Table S7: the IncRNAs, mARNAs, and miRNAs in the ceRNA network. [file 8735551.f2.zip › Table S5.pdf]

Table S5. 96 DEmiRNAs between PTC and normal thyroid tissues.

| DEmiRNAs | logFC | logCPM | PValue | FDR |
|---|---|---|---|---|
| hsa-mir-34a | 2.28911493 | 8.879361205 | 5.82E-68 | 3.86E-65 |
| hsa-mir-4709 | 3.14010781 | 3.465177241 | 1.47E-67 | 4.89E-65 |
| hsa-mir-221 | 3.36588369 | 10.80226033 | 1.92E-60 | 4.24E-58 |
| hsa-mir-146b | 5.7702319 | 14.73464122 | 4.98E-58 | 8.26E-56 |
| hsa-mir-222 | 3.0282631 | 8.861547112 | 9.01E-57 | 1.20E-54 |
| hsa-mir-144 | -2.1468106 | 8.243203227 | 1.63E-50 | 1.80E-48 |
| hsa-mir-181a-2 | 1.5375399 | 12.92848346 | 6.66E-49 | 6.32E-47 |
| hsa-mir-451a | -2.126168 | 10.37394843 | 4.51E-48 | 3.75E-46 |
| hsa-mir-181b-1 | 1.54973554 | 9.276822715 | 7.62E-48 | 5.62E-46 |
| hsa-mir-181b-2 | 1.59331483 | 9.268591888 | 1.20E-46 | 7.97E-45 |
| hsa-mir-551b | 4.51705156 | 5.104130236 | 2.55E-44 | 1.54E-42 |
| hsa-mir-486-2 | -2.062216 | 7.354848273 | 1.43E-39 | 7.31E-38 |
| hsa-mir-486-1 | -2.0583663 | 7.361697631 | 1.59E-39 | 7.56E-38 |
| hsa-mir-21 | 1.89921408 | 17.53736501 | 2.55E-34 | 1.13E-32 |
| hsa-mir-6854 | 1.86349817 | 0.867300271 | 1.27E-30 | 5.27E-29 |
| hsa-mir-6860 | 4.77792908 | 0.095847992 | 2.37E-30 | 9.26E-29 |
| hsa-mir-1258 | -2.456521 | -0.046304364 | 1.56E-26 | 5.16E-25 |
| hsa-mir-6730 | 2.21979959 | 0.559695522 | 4.24E-25 | 1.28E-23 |
| hsa-mir-7156 | 4.98580537 | 0.886637251 | 6.63E-24 | 1.92E-22 |
| hsa-mir-375 | -2.898651 | 12.72708162 | 1.10E-23 | 3.05E-22 |
| hsa-mir-934 | 4.71912176 | 1.054305109 | 2.53E-23 | 6.73E-22 |
| hsa-mir-4732 | -1.8593108 | 0.294162028 | 4.17E-23 | 1.07E-21 |
| hsa-mir-187 | 3.17410138 | 3.431453103 | 3.68E-21 | 8.72E-20 |
| hsa-mir-508 | 1.93622032 | 5.685386514 | 4.84E-20 | 1.06E-18 |
| hsa-mir-31 | 2.45314721 | 6.662864541 | 8.69E-20 | 1.70E-18 |
| hsa-mir-514a-3 | 2.03763954 | 3.034398211 | 5.09E-19 | 9.38E-18 |
| hsa-mir-509-3 | 2.11199764 | 2.34388876 | 1.35E-18 | 2.19E-17 |
| hsa-mir-509-2 | 1.99528243 | 1.985210705 | 8.61E-18 | 1.27E-16 |
| hsa-mir-509-1 | 2.02392835 | 2.011232875 | 1.22E-17 | 1.76E-16 |
| hsa-mir-514a-1 | 1.90606339 | 3.020536042 | 1.79E-17 | 2.48E-16 |
| hsa-mir-514a-2 | 1.88571852 | 3.035020586 | 5.28E-17 | 7.16E-16 |
| hsa-mir-3189 | 3.0452231 | -0.783720286 | 6.65E-16 | 8.17E-15 |
| hsa-mir-3065 | 1.54078698 | 7.524432477 | 3.17E-15 | 3.63E-14 |
| hsa-mir-503 | 1.55051003 | 4.928725094 | 1.49E-14 | 1.62E-13 |
| hsa-mir-513c | 2.51880037 | -0.52101433 | 1.26E-13 | 1.27E-12 |
| hsa-mir-506 | 2.03618837 | 0.223481515 | 7.24E-13 | 6.68E-12 |
| hsa-mir-1179 | -1.6613531 | 2.847230585 | 1.17E-12 | 1.03E-11 |
| hsa-mir-196a-2 | 3.47112529 | 2.061130087 | 4.63E-12 | 3.85E-11 |
| hsa-mir-184 | 3.62091101 | 2.922621585 | 6.05E-12 | 4.96E-11 |
| hsa-mir-196a-1 | 3.35683493 | 1.875474179 | 3.89E-11 | 2.84E-10 |
| hsa-mir-873 | -1.8364113 | 0.756733581 | 9.93E-11 | 6.87E-10 |
| hsa-mir-891a | 3.4946429 | 7.590427854 | 1.57E-10 | 1.05E-09 |
| hsa-mir-4758 | 1.95299571 | -0.7906426 | 2.17E-10 | 1.42E-09 |
| hsa-mir-3150b | -1.6991716 | -0.601535115 | 3.66E-10 | 2.32E-09 |
| hsa-mir-876 | -1.6786353 | -0.428757195 | 5.27E-10 | 3.27E-09 |
| hsa-mir-6499 | 2.48523708 | -0.813154152 | 3.30E-09 | 1.82E-08 |
| hsa-mir-372 | 3.46735637 | 1.388177228 | 4.82E-09 | 2.56E-08 |
| hsa-mir-514b | 1.89609162 | -0.594832693 | 8.68E-09 | 4.47E-08 |
| hsa-mir-147b | 1.70485315 | 0.06300342 | 1.05E-08 | 5.34E-08 |
| hsa-mir-519a-1 | 7.42338837 | 4.170741308 | 1.54E-08 | 7.75E-08 |
| hsa-mir-767 | 2.67978691 | -0.60928365 | 3.00E-08 | 1.42E-07 |
| hsa-mir-205 | 2.08616455 | 7.449823649 | 1.12E-07 | 4.94E-07 |
| hsa-mir-526b | 6.06966385 | 4.545532626 | 1.35E-07 | 5.88E-07 |
| hsa-mir-512-1 | 5.75139857 | 1.617656811 | 1.44E-07 | 6.23E-07 |
| hsa-mir-516b-2 | 5.3678626 | 0.685192295 | 1.87E-07 | 7.96E-07 |
| hsa-mir-512-2 | 5.53732647 | 1.619687464 | 1.90E-07 | 8.03E-07 |

| | | | | |
|---|---|---|---|---|
| hsa-mir-516b-1 | 6.00331543 | 1.830070813 | 2.11E-07 | 8.74E-07 |
| hsa-mir-516a-1 | 5.68574592 | 2.039340703 | 4.38E-07 | 1.74E-06 |
| hsa-mir-518b | 5.43720815 | 2.259943365 | 4.55E-07 | 1.80E-06 |
| hsa-mir-520g | 5.35682928 | 1.066537116 | 5.00E-07 | 1.94E-06 |
| hsa-mir-498 | 4.00213042 | -0.581618379 | 6.17E-07 | 2.34E-06 |
| hsa-mir-516a-2 | 5.64444305 | 2.001116562 | 8.67E-07 | 3.23E-06 |
| hsa-mir-483 | 2.43192913 | 2.868245756 | 9.72E-07 | 3.57E-06 |
| hsa-mir-1305 | 1.55070137 | -0.642839401 | 1.05E-06 | 3.83E-06 |
| hsa-mir-515-2 | 4.41348764 | -0.102525705 | 1.31E-06 | 4.65E-06 |
| hsa-mir-520a | 5.33971284 | 3.117106693 | 1.46E-06 | 5.12E-06 |
| hsa-mir-515-1 | 4.4442218 | -0.068662849 | 1.78E-06 | 6.05E-06 |
| hsa-mir-518f | 5.51152989 | 1.965819197 | 1.98E-06 | 6.66E-06 |
| hsa-mir-520h | 4.72624654 | 0.42969282 | 2.20E-06 | 7.30E-06 |
| hsa-mir-518a-2 | 4.84341982 | 0.716927588 | 2.25E-06 | 7.42E-06 |
| hsa-mir-517c | 5.01681881 | 0.825049992 | 2.34E-06 | 7.71E-06 |
| hsa-mir-518c | 5.06229822 | 2.105908296 | 2.58E-06 | 8.27E-06 |
| hsa-mir-1323 | 4.8796823 | 1.146256613 | 2.66E-06 | 8.45E-06 |
| hsa-mir-519d | 4.74011886 | 0.830064502 | 3.73E-06 | 1.16E-05 |
| hsa-mir-525 | 4.76672138 | 1.787193396 | 3.97E-06 | 1.23E-05 |
| hsa-mir-520b | 4.95332953 | 1.65958134 | 5.49E-06 | 1.67E-05 |
| hsa-mir-522 | 4.82565568 | 1.023184931 | 6.01E-06 | 1.80E-05 |
| hsa-mir-892a | 3.22991165 | 2.254017325 | 7.49E-06 | 2.22E-05 |
| hsa-mir-520d | 4.25988784 | 0.209118936 | 9.28E-06 | 2.71E-05 |
| hsa-mir-519a-2 | 4.85009808 | 2.080489662 | 1.00E-05 | 2.90E-05 |
| hsa-mir-518e | 4.43876399 | 0.917784313 | 1.04E-05 | 2.99E-05 |
| hsa-mir-519c | 4.38563505 | 0.410393252 | 1.58E-05 | 4.42E-05 |
| hsa-mir-523 | 3.9387052 | -0.123856525 | 2.70E-05 | 7.28E-05 |
| hsa-mir-892b | 3.33406728 | 0.011593231 | 2.98E-05 | 7.93E-05 |
| hsa-mir-517a | 4.38365208 | 2.457445414 | 2.99E-05 | 7.95E-05 |
| hsa-mir-517b | 4.3906147 | 2.453564135 | 3.01E-05 | 7.97E-05 |
| hsa-mir-527 | 4.06279593 | 0.433380681 | 3.06E-05 | 8.05E-05 |
| hsa-mir-524 | 3.60120884 | -0.589419116 | 3.14E-05 | 8.23E-05 |
| hsa-mir-373 | 2.55449865 | -0.480539685 | 3.35E-05 | 8.65E-05 |
| hsa-mir-888 | 3.10045405 | 0.882928792 | 3.73E-05 | 9.60E-05 |
| hsa-mir-518a-1 | 3.92938691 | 0.324286901 | 4.32E-05 | 0.00011043 |
| hsa-mir-1283-2 | 3.27495849 | -0.693265964 | 8.94E-05 | 0.00021746 |
| hsa-mir-891b | 2.61617122 | 0.706691958 | 0.0001 | 0.00024884 |
| hsa-mir-892c | 3.11843546 | -0.158714488 | 0.00014 | 0.00034426 |
| hsa-mir-520f | 3.3431357 | -0.178181419 | 0.00022 | 0.00050955 |
| hsa-mir-1269a | 1.8533748 | -0.847902647 | 0.00051 | 0.001101 |
