# Supplementary material for: A Potential Four-Gene Signature and Nomogram for Predicting the Overall Survival of Papillary Thyroid Cancer
Source: Dis Markers. 2022 Aug 30;2022:8735551. doi: 10.1155/2022/8735551 (PMC9526076; doi:10.1155/2022/8735551)
Supplement: Supplementary 2 — Table S1: details of the GEO and TCGA datasets used in this study. Table S2: samples in HPA database. Table S3: the sequences of primers. Table S4: univariate Cox regression of the 176 genes in the training cohort. Table S5: 96 DEmiRNAs between PTC and normal thyroid tissues. Table S6: 839 DEIncRNAs between PTC and normal thyroid tissues. Table S7: the IncRNAs, mARNAs, and miRNAs in the ceRNA network. [file 8735551.f2.zip › Table S6.pdf]

Table S6. 839 DElncRNAs between PTC and normal thyroid tissues.

DElncRNAs	logFC	logCPM	PValue	FDR
AL157714.2	7.263436	10.2018	6.50E-47	1.97E-44
AP002358.1	6.431162	8.895576	1.34E-32	8.10E-31
AC016717.2	6.25873	7.730051	2.75E-23	6.42E-22
AC079630.2	6.190273	10.44721	1.18E-38	1.46E-36
LINC02461	5.762469	4.74863	8.36E-17	9.83E-16
AC025419.1	5.674921	7.720355	3.19E-52	1.51E-49
LINC02408	5.520816	7.791268	9.15E-36	7.97E-34
LINC02471	5.432068	9.876895	2.49E-50	9.93E-48
UNC5B-AS1	5.160284	6.550444	2.78E-52	1.41E-49
NPSR1-AS1	5.135339	4.038671	4.32E-16	4.67E-15
AC012038.2	5.132077	5.328607	1.63E-42	3.06E-40
AC104257.1	5.075711	3.864733	2.75E-22	5.86E-21
AC007255.1	5.048033	8.805316	3.71E-41	5.98E-39
OPCML-IT1	5.028654	3.163982	1.61E-13	1.33E-12
AL035409.1	4.989261	4.481663	4.83E-23	1.11E-21
AC013457.1	4.950499	3.997105	1.74E-10	9.84E-10
AC011473.3	4.944425	3.18164	1.17E-20	2.10E-19
AC103726.2	4.933261	3.059625	1.25E-19	2.02E-18
AC254633.1	4.933198	9.671906	1.45E-43	3.14E-41
AC104024.2	4.927786	5.450914	6.23E-29	2.86E-27
LINC02082	4.894789	5.723967	1.57E-38	1.85E-36
AC244502.3	4.837783	5.51284	2.81E-25	8.58E-24
LINC02454	4.803937	7.39563	3.17E-54	2.00E-51
AL355312.3	4.799638	6.395079	4.05E-51	1.80E-48
AC008063.1	4.725536	5.556433	2.34E-40	3.41E-38
AL109754.1	4.697061	4.766044	6.76E-21	1.23E-19
AC096996.2	4.624361	3.204991	1.08E-14	1.01E-13
AC006450.3	4.576263	3.323744	4.67E-06	1.47E-05
LINC01510	4.504414	5.215859	9.29E-27	3.30E-25
AL022314.1	4.494827	3.244946	2.22E-18	3.09E-17
AC023490.3	4.491475	5.781293	6.47E-26	2.09E-24
LINC01969	4.479359	2.843265	1.49E-27	5.71E-26
AC104574.2	4.478884	6.179539	4.60E-13	3.62E-12
AC016405.3	4.43448	7.803627	1.15E-40	1.77E-38
AC243585.1	4.433125	3.638927	2.72E-22	5.82E-21
AC092954.1	4.391032	4.967343	2.20E-30	1.17E-28
FAM170B-AS1	4.375741	4.305785	1.45E-28	6.33E-27
AC244502.1	4.375506	8.242863	1.50E-23	3.59E-22
AC090023.1	4.355396	2.784726	1.48E-25	4.71E-24
LRP4-AS1	4.353685	6.271806	2.49E-48	8.57E-46
AL590644.1	4.337938	3.401624	6.44E-17	7.74E-16
TMEM108-AS1	4.329612	4.545015	5.37E-18	7.22E-17
LINC01660	4.326314	2.815172	4.25E-17	5.23E-16
LINC01614	4.326061	5.525308	1.25E-22	2.75E-21
AP005233.2	4.284232	4.312451	2.59E-21	4.91E-20
AC007731.1	4.235103	3.275389	2.09E-12	1.51E-11
HOXA11-AS	4.218518	3.968537	1.39E-13	1.16E-12
CLDN10-AS1	4.217975	3.497582	3.46E-13	2.76E-12
AC007614.4	4.205754	5.21252	4.14E-20	7.00E-19
AL359313.1	4.202777	2.809861	1.26E-13	1.05E-12
AC092954.2	4.201106	3.504474	1.39E-27	5.43E-26
AL133343.3	4.174814	3.399293	2.03E-18	2.84E-17
AC016405.2	4.159647	5.640538	9.28E-32	5.25E-30
LINC01170	4.152422	5.16416	1.57E-37	1.72E-35
FLJ16779	4.117398	7.5632	6.78E-22	1.37E-20
AC097478.1	4.116395	8.330982	1.23E-17	1.59E-16

AC079630.1	4.09204	11.0662	2.05E-47	6.46E-45
FP236383.3	4.090398	2.987195	7.65E-06	2.31E-05
AC012313.4	4.090241	7.729414	1.97E-42	3.55E-40
AC090023.2	4.066645	4.004888	3.30E-34	2.43E-32
FAM230B	4.059538	4.038676	1.58E-26	5.44E-25
AC107308.1	4.055849	4.175092	1.98E-36	2.00E-34
HAGLROS	4.054732	5.93054	1.65E-42	3.06E-40
AC091138.1	4.038226	3.816028	4.63E-13	3.64E-12
IGFL2-AS1	4.03165	3.967422	4.12E-22	8.62E-21
AC243773.2	4.006558	3.206237	1.38E-05	4.00E-05
LINC02257	4.001476	3.675412	2.34E-17	2.96E-16
AC006262.1	3.993864	6.188006	3.19E-24	8.24E-23
LINC02349	3.98008	2.576411	1.73E-11	1.11E-10
LINC01977	3.968767	6.801509	7.45E-77	1.88E-73
LINC00284	3.949236	5.981331	5.75E-29	2.67E-27
LINC02306	3.940598	2.579346	2.43E-09	1.20E-08
AC002401.4	3.928341	7.204833	3.61E-29	1.72E-27
LINC02350	3.927941	2.2914	1.24E-16	1.43E-15
IGF2-AS	3.903754	5.544765	1.29E-07	5.07E-07
AL132709.1	3.89052	3.072519	1.44E-16	1.65E-15
LINC01460	3.86794	4.551019	2.30E-34	1.71E-32
AC080038.1	3.845288	7.282507	1.75E-44	4.28E-42
AC005725.1	3.828819	7.090701	5.08E-29	2.39E-27
AC006262.2	3.779961	3.636885	8.30E-19	1.21E-17
AL137026.1	3.777345	7.802602	2.32E-24	6.16E-23
AC011473.1	3.771644	2.597871	4.73E-12	3.26E-11
AL354863.1	3.724828	2.444057	1.04E-06	3.60E-06
AP001999.3	3.67837	2.307545	1.15E-06	3.93E-06
AC018618.1	3.672519	3.468623	1.35E-12	9.99E-12
AC012462.3	3.667248	2.797674	9.55E-24	2.34E-22
AC012668.3	3.664242	4.758687	2.99E-30	1.56E-28
LINC01705	3.659939	3.750932	7.12E-10	3.76E-09
GDNF-AS1	3.659794	2.937149	8.20E-13	6.17E-12
LINC00460	3.625835	3.814136	2.62E-19	4.04E-18
TMEM92-AS1	3.603509	3.763999	2.45E-25	7.61E-24
MKX-AS1	3.602555	3.42724	2.20E-12	1.59E-11
AC093895.1	3.598242	3.366232	2.42E-24	6.42E-23
AL391845.2	3.594098	5.421479	9.44E-60	8.94E-57
AP000552.1	3.591268	2.454491	1.02E-19	1.67E-18
LINC01747	3.590783	5.181139	3.33E-28	1.39E-26
AP006565.1	3.588324	3.634345	1.30E-15	1.35E-14
AC068987.1	3.580132	4.632735	7.87E-27	2.83E-25
HAGLR	3.572504	9.171764	1.78E-26	6.08E-25
UCA1	3.571907	4.885861	6.71E-12	4.56E-11
LINC00973	3.570245	5.069795	1.28E-16	1.47E-15
AC034206.1	3.544471	3.962218	1.50E-11	9.82E-11
LINC00836	3.509393	3.356158	8.08E-09	3.70E-08
LINC02188	3.502964	4.961311	8.33E-14	7.10E-13
AC007614.1	3.493655	2.985962	5.27E-14	4.58E-13
AL512785.1	3.49182	2.101865	2.33E-11	1.47E-10
LINC00511	3.489517	8.280337	2.24E-30	1.19E-28
LINC01204	3.485708	4.439086	2.09E-16	2.35E-15
AC084262.1	3.481328	3.391476	5.93E-29	2.74E-27
LINC01021	3.469742	4.542036	2.14E-15	2.18E-14
AC007207.2	3.466275	6.862253	6.12E-46	1.60E-43
AL591806.1	3.457458	2.700402	4.81E-26	1.59E-24
LINC02343	3.457004	3.772218	1.49E-22	3.26E-21
AL593854.1	3.443133	2.301069	1.10E-16	1.28E-15

LINC00475	3.430459	6.507722	8.98E-37	9.32E-35
LINC01662	3.430114	2.00186	8.38E-11	4.91E-10
AL355512.1	3.394945	5.292346	1.17E-33	7.96E-32
AL354824.1	3.382353	2.160605	3.82E-17	4.73E-16
LINC00302	3.37733	1.989423	1.44E-07	5.61E-07
AL096865.1	3.364218	6.592691	2.37E-26	8.06E-25
AC069120.1	3.362481	3.837561	1.64E-21	3.19E-20
AP000851.2	3.357747	5.00595	4.69E-09	2.23E-08
AC116345.1	3.355795	3.589465	5.38E-10	2.87E-09
AF131215.3	3.340897	3.342025	3.08E-24	7.99E-23
AC023301.1	3.338611	6.733317	4.43E-17	5.44E-16
AC012625.1	3.323533	2.978818	7.14E-17	8.50E-16
LINC01231	3.320553	2.097376	1.80E-06	6.02E-06
AC022784.2	3.309654	3.14257	3.52E-09	1.70E-08
LINC00704	3.306512	3.096783	1.48E-12	1.09E-11
AC018816.1	3.304493	8.105058	4.71E-50	1.78E-47
AP000997.2	3.304374	3.386161	1.28E-30	6.86E-29
AC011338.1	3.281156	7.51447	2.19E-56	1.66E-53
AL354919.2	3.275086	2.742787	1.02E-15	1.07E-14
AL139042.1	3.272113	2.001105	2.36E-10	1.31E-09
AC015909.1	3.271861	2.837038	1.33E-20	2.35E-19
KCNMB2-AS1	3.269149	3.622549	1.06E-29	5.23E-28
LINC01770	3.267969	7.179698	2.56E-43	5.39E-41
AC026355.2	3.261383	2.306866	4.78E-13	3.74E-12
AC025580.1	3.256201	7.383006	2.63E-25	8.09E-24
AC069120.3	3.248957	3.124025	1.34E-23	3.22E-22
NR2F1-AS1	3.242696	10.38711	2.07E-36	2.05E-34
LINC01293	3.24097	4.699515	4.60E-09	2.19E-08
AL158166.1	3.217996	4.895633	8.24E-38	9.40E-36
AL353581.1	3.217096	2.638154	4.68E-08	1.93E-07
AC022148.1	3.213731	6.633783	4.25E-28	1.73E-26
LAMP5-AS1	3.202963	4.64804	1.74E-07	6.70E-07
LBX1-AS1	3.197926	2.516834	0.000117609	0.00029592
AP000696.1	3.195867	2.1396	3.91E-05	0.00010615
LINC00423	3.185653	2.945799	1.81E-20	3.17E-19
AC097468.1	3.178843	2.871178	2.09E-21	4.03E-20
AP002957.1	3.176116	3.962791	1.41E-26	4.89E-25
AC004847.1	3.16625	9.152484	2.73E-16	3.01E-15
AC144833.1	3.144006	2.580144	4.86E-09	2.31E-08
AC100872.1	3.139656	2.160241	8.85E-12	5.94E-11
AC073284.1	3.125499	2.06621	8.23E-10	4.32E-09
AC245041.2	3.120972	2.814977	9.01E-10	4.69E-09
AC016205.1	3.118744	5.534192	8.06E-33	5.01E-31
LINC02345	3.115359	5.814593	2.85E-25	8.65E-24
LINC01711	3.114817	4.212746	4.22E-16	4.56E-15
AC011944.1	3.113161	5.911738	1.27E-20	2.27E-19
LINC02407	3.102004	4.072367	3.30E-17	4.11E-16
AC245041.3	3.087463	4.284223	2.69E-13	2.16E-12
TM4SF1-AS1	3.085076	5.846399	5.05E-16	5.43E-15
AC112176.1	3.080366	3.749864	1.53E-13	1.26E-12
AC107959.3	3.079019	4.134674	1.91E-32	1.15E-30
AC090673.1	3.07084	9.51513	2.93E-41	4.94E-39
AL138900.3	3.070774	4.4128	6.57E-11	3.92E-10
AL391807.1	3.070392	6.894179	5.11E-28	2.04E-26
AC025253.1	3.060521	2.724069	2.02E-14	1.83E-13
MIR31HG	3.049395	7.663327	2.46E-24	6.50E-23
AL645608.9	3.046647	4.098275	1.32E-21	2.58E-20
SYNPR-AS1	3.045876	2.970237	4.04E-08	1.68E-07

AC006249.1	3.018326	3.375203	2.93E-23	6.81E-22
AC023886.1	3.014913	2.89323	2.26E-07	8.56E-07
AC092436.2	3.013715	2.206014	4.26E-18	5.77E-17
AC008649.1	2.99828	3.923566	7.61E-15	7.28E-14
AC116021.1	2.991357	3.118248	6.65E-29	3.03E-27
LINC01561	2.988595	3.048552	8.50E-10	4.45E-09
AP000997.1	2.973909	3.219243	9.81E-34	6.76E-32
AL158166.2	2.972952	3.886204	4.33E-30	2.22E-28
AC097478.2	2.972765	3.725141	9.38E-10	4.87E-09
AC006159.2	2.972671	4.194544	7.95E-18	1.06E-16
LINC01788	2.967699	2.566332	8.64E-19	1.25E-17
AC063926.1	2.957714	3.985769	9.54E-13	7.14E-12
AL592528.1	2.955109	2.520579	5.43E-07	1.95E-06
AC010336.5	2.945357	3.192635	4.50E-20	7.57E-19
AC097478.3	2.926626	3.215386	5.25E-09	2.48E-08
AP002991.1	2.923283	3.325858	8.62E-32	4.91E-30
AP001029.1	2.911884	4.916513	7.32E-28	2.89E-26
AC099066.2	2.90642	3.222904	1.64E-14	1.50E-13
LINC00457	2.895557	3.124202	1.65E-11	1.07E-10
LINC01896	2.894331	4.110825	0.00167049	0.00344277
LINC00607	2.878593	7.270991	5.54E-27	2.01E-25
AC090568.2	2.877881	3.339072	5.37E-19	8.06E-18
DUXAP8	2.854801	6.339138	2.68E-23	6.28E-22
LINC01878	2.853124	5.038793	9.04E-18	1.19E-16
HCG22	2.852868	8.818652	2.08E-19	3.27E-18
LINC02347	2.849848	3.315905	3.84E-10	2.09E-09
AC120498.4	2.834918	2.846381	2.12E-08	9.09E-08
AP003174.1	2.830985	3.805683	1.19E-39	1.61E-37
LINC00922	2.807155	2.761803	4.22E-09	2.02E-08
BX276092.9	2.797708	6.069257	1.28E-28	5.64E-27
Z98257.1	2.796381	4.576855	1.34E-23	3.22E-22
AL831784.1	2.795106	2.783544	0.000402797	0.00092557
TNRC6C-AS1	2.785339	10.9147	1.31E-41	2.26E-39
LINC01483	2.783326	5.783138	3.46E-29	1.66E-27
AL139158.2	2.780586	3.778565	4.32E-21	8.00E-20
AC093281.2	2.77887	3.118973	3.75E-22	7.92E-21
AL691482.3	2.776384	4.523496	3.09E-15	3.07E-14
LINC02332	2.775584	3.770358	2.09E-14	1.89E-13
AL596218.1	2.770522	2.953412	3.88E-14	3.42E-13
AC110285.3	2.767748	4.864667	1.47E-26	5.08E-25
LL22NC03-63E9.3	2.744989	2.45674	0.000131895	0.00032956
LINC01612	2.744664	2.180515	1.88E-05	5.35E-05
AP001029.2	2.73609	6.777692	2.27E-35	1.93E-33
AC068858.1	2.729827	3.940295	6.97E-21	1.27E-19
UG0898H09	2.724226	5.087357	2.97E-06	9.56E-06
AL391597.1	2.697575	6.426856	1.11E-22	2.46E-21
AC068594.1	2.69691	4.099449	2.09E-36	2.05E-34
AC004540.2	2.692837	5.13628	1.43E-08	6.33E-08
AL365356.5	2.692406	2.697119	1.13E-11	7.49E-11
LINC02450	2.683543	2.566475	9.78E-10	5.07E-09
LINC01933	2.68173	5.774562	2.76E-12	1.96E-11
MIR205HG	2.673696	7.022733	9.78E-10	5.07E-09
LINC00620	2.672559	2.01587	9.13E-06	2.72E-05
LINC00887	2.670706	7.558835	9.23E-22	1.83E-20
LINC01267	2.659047	3.730318	6.54E-25	1.91E-23
AC073316.3	2.656986	3.923741	4.65E-12	3.21E-11
LINC02458	2.639735	4.06845	9.96E-18	1.30E-16
AC008609.1	2.639048	2.028329	2.04E-09	1.02E-08

AL138876.1	2.638723	2.313895	5.87E-17	7.08E-16
CDKN2B-AS1	2.638398	5.121234	3.02E-25	9.07E-24
AC099560.1	2.630508	3.394262	8.94E-10	4.66E-09
LINC02159	2.62519	5.452296	2.49E-14	2.23E-13
LINC02303	2.612178	2.533133	8.62E-07	3.01E-06
AC136618.1	2.608423	1.991422	4.92E-14	4.28E-13
LINC01918	2.601772	5.26861	3.71E-27	1.38E-25
AC007368.1	2.595629	1.994914	9.80E-09	4.43E-08
AC005392.2	2.588951	2.951761	4.16E-10	2.24E-09
AC007785.1	2.581847	2.617116	3.04E-14	2.69E-13
AC004233.4	2.577504	4.191663	8.43E-33	5.19E-31
AC090796.1	2.568833	2.018403	7.70E-09	3.54E-08
AC126773.2	2.567123	6.566895	8.62E-47	2.51E-44
AC087627.1	2.566056	2.514888	6.67E-16	7.12E-15
AP000679.1	2.563336	3.399416	2.20E-19	3.44E-18
AC025284.1	2.559938	2.47585	5.00E-10	2.68E-09
AP000525.1	2.559657	3.090109	7.71E-17	9.14E-16
AL161716.1	2.553675	2.518221	1.85E-13	1.51E-12
LINC00707	2.552756	3.496367	1.74E-08	7.60E-08
DCTN1-AS1	2.552013	4.530448	9.44E-27	3.34E-25
AC009229.2	2.551348	3.428424	6.91E-32	4.00E-30
AC026368.1	2.549355	5.58875	1.22E-38	1.50E-36
AC134312.5	2.548266	4.443146	2.51E-09	1.23E-08
AC092920.1	2.545174	2.87266	1.21E-24	3.41E-23
LINC01208	2.543019	2.877004	3.03E-21	5.71E-20
LINC02154	2.535416	3.711772	2.83E-09	1.38E-08
LINC00365	2.533359	4.286706	1.08E-14	1.01E-13
AC018541.1	2.532119	2.667991	1.16E-07	4.57E-07
AC141928.1	2.526691	6.85341	5.34E-15	5.18E-14
HOTAIR	2.520985	2.024571	1.83E-06	6.10E-06
AL139420.1	2.519742	1.998872	3.95E-08	1.65E-07
WASIR1	2.515496	2.425468	8.13E-06	2.44E-05
LINC00514	2.502493	5.4705	1.06E-38	1.34E-36
AC011483.2	2.501331	2.346689	1.03E-07	4.08E-07
AP000851.1	2.487719	5.652544	1.07E-07	4.23E-07
MEG9	2.48644	3.871715	1.80E-11	1.15E-10
AP007216.2	2.478182	3.216336	1.72E-24	4.71E-23
AC015921.1	2.475978	5.37057	1.31E-43	2.92E-41
AP001995.2	2.470988	2.457922	6.48E-18	8.64E-17
AC012213.4	2.45559	5.095178	5.91E-23	1.35E-21
AL138902.1	2.443198	2.173965	2.44E-12	1.74E-11
AC026369.1	2.443062	4.917161	6.64E-16	7.10E-15
AC005479.2	2.442729	6.706754	4.02E-29	1.90E-27
AC009549.1	2.434689	7.858842	1.02E-24	2.89E-23
PICSAR	2.428053	3.117774	2.96E-18	4.05E-17
AL844175.1	2.423633	2.050568	1.04E-06	3.59E-06
MYO16-AS1	2.420653	2.258972	8.69E-06	2.60E-05
AC005479.3	2.420421	6.892935	2.17E-36	2.11E-34
AL135999.3	2.4185	4.739248	2.09E-10	1.17E-09
AC100803.3	2.413658	5.380907	3.30E-30	1.71E-28
AC011373.1	2.412279	2.627217	4.12E-19	6.23E-18
AL713998.1	2.402398	3.145475	4.63E-05	0.0001244
AC011383.1	2.402267	3.215548	5.65E-26	1.84E-24
AC010336.2	2.397505	3.385698	3.56E-28	1.46E-26
AP001505.1	2.390728	2.591989	2.51E-09	1.24E-08
CYP4A22-AS1	2.389585	3.852832	7.29E-32	4.19E-30
AC079210.1	2.378807	4.014473	3.93E-16	4.26E-15
AC087273.2	2.371922	2.775265	4.71E-15	4.62E-14

AL353681.1	2.365711	4.377094	3.45E-09	1.67E-08
AC093865.1	2.36328	3.976368	6.89E-22	1.39E-20
FALEC	2.352673	4.946171	3.91E-11	2.40E-10
AC040160.1	2.347644	7.13193	8.05E-53	4.36E-50
AC007099.1	2.344461	2.267768	3.14E-13	2.51E-12
AC009041.2	2.343588	9.489217	2.27E-19	3.54E-18
AL645608.1	2.33703	9.073008	7.43E-21	1.35E-19
AC018450.1	2.333491	5.616483	1.05E-28	4.70E-27
AL161733.1	2.331224	2.104592	2.65E-08	1.12E-07
CYP1B1-AS1	2.330314	7.331019	1.51E-34	1.15E-32
LINC01141	2.327897	2.689271	2.99E-16	3.29E-15
DPP10-AS1	2.324969	4.758037	7.39E-09	3.40E-08
AC000067.1	2.321653	3.183821	8.84E-18	1.17E-16
TYMSOS	2.319757	5.660495	1.04E-32	6.35E-31
AC093821.1	2.310285	2.048547	7.57E-11	4.48E-10
LINC02212	2.308527	2.110446	0.002127121	0.00429965
AC004540.1	2.303547	6.255907	2.87E-30	1.51E-28
AC091020.1	2.302013	3.39376	2.67E-09	1.31E-08
ABHD11-AS1	2.298004	8.190938	2.34E-18	3.24E-17
MGAT3-AS1	2.292988	3.359091	4.72E-14	4.12E-13
LINC01732	2.290344	2.228753	1.33E-14	1.23E-13
AC073316.2	2.283171	2.136976	1.85E-06	6.16E-06
AL512422.2	2.279122	4.340034	2.29E-13	1.84E-12
LINC02420	2.272613	2.500547	1.34E-07	5.25E-07
AC016831.6	2.272597	2.837564	2.30E-07	8.70E-07
LINC00870	2.271899	3.071331	0.001218701	0.00257902
VAC14-AS1	2.250748	5.022099	8.39E-20	1.39E-18
AC113383.1	2.246843	3.996746	1.07E-22	2.37E-21
AP002754.1	2.242156	4.974748	7.25E-19	1.06E-17
LINC00974	2.240974	2.26277	0.001861488	0.00380638
TCERG1L-AS1	2.235537	5.151085	4.19E-24	1.06E-22
AC019117.2	2.231046	4.434473	3.44E-09	1.67E-08
AC078950.1	2.22664	2.671512	0.000112347	0.00028324
AL353150.1	2.220638	6.771472	2.94E-35	2.39E-33
AP000695.1	2.219838	3.70205	4.48E-16	4.83E-15
LINC00313	2.218883	2.45276	2.99E-11	1.87E-10
SFTA1P	2.217531	6.168381	1.01E-18	1.44E-17
AC120036.5	2.212646	7.43496	2.47E-35	2.08E-33
MIR181A2HG	-2.20348	8.431237	4.20E-42	7.40E-40
OVAAL	2.203113	2.085224	8.62E-09	3.93E-08
LINC01858	2.197985	2.225387	4.12E-12	2.88E-11
LINC01608	2.197681	1.96639	0.000781034	0.00171461
AC012150.2	2.196579	2.668385	1.49E-07	5.78E-07
AC009229.1	2.19237	2.695689	1.53E-16	1.74E-15
DOCK9-AS2	2.182919	9.864912	1.49E-44	3.75E-42
LINC00113	2.178493	4.277244	4.05E-12	2.83E-11
PP14571	2.177818	6.261144	7.84E-23	1.76E-21
AL365356.4	2.17694	2.259942	3.72E-08	1.56E-07
AC006450.2	2.175642	2.158767	8.96E-05	0.0002294
PSG8-AS1	2.173231	2.317234	2.10E-07	8.02E-07
AP000251.1	2.172493	3.880233	8.50E-19	1.23E-17
AL162511.1	2.171951	7.991783	8.92E-29	4.02E-27
AC006487.1	2.171691	2.913275	8.36E-12	5.63E-11
RUNDC3A-AS1	2.171547	9.241616	4.00E-67	7.58E-64
AC008622.2	2.169011	4.240653	1.52E-20	2.69E-19
AL356311.1	2.167841	2.063377	2.21E-05	6.22E-05
AL365259.1	2.164986	7.099569	3.50E-23	8.06E-22
LINC01426	2.164557	6.429604	7.49E-22	1.51E-20

TEX26-AS1	2.163534	5.193306	4.76E-13	3.73E-12
HOXC-AS2	2.156273	2.588439	2.63E-06	8.53E-06
AL022334.2	2.154752	2.171387	4.77E-10	2.56E-09
SMIM2-IT1	2.153629	2.23721	5.89E-08	2.41E-07
AC023043.4	2.153586	4.376921	3.58E-32	2.11E-30
KCNJ2-AS1	2.148282	8.667331	4.03E-36	3.81E-34
AC107057.1	2.146828	4.559791	5.57E-15	5.40E-14
LINC02160	2.145458	2.544532	6.90E-05	0.00018094
AC116021.2	2.143253	2.160081	0.006174162	0.01136706
AL645608.3	2.141299	4.019535	1.34E-11	8.77E-11
AC110015.1	2.138958	2.292693	4.71E-11	2.86E-10
AL590428.1	2.134588	4.671512	1.37E-16	1.57E-15
AC107294.2	2.131129	6.923562	2.23E-29	1.08E-27
AL136298.1	2.130231	2.636017	7.22E-08	2.92E-07
AC025580.2	2.129897	4.466794	5.95E-15	5.73E-14
AC137932.2	2.128293	4.297947	7.04E-34	4.89E-32
LINC02003	2.127414	2.302302	4.86E-07	1.76E-06
LINC01016	2.125982	3.333918	0.000281389	0.00066246
AL731684.1	2.11757	2.24615	1.06E-11	7.03E-11
AP003481.1	2.115763	3.334516	1.25E-12	9.26E-12
AC055733.2	2.113427	2.03951	1.38E-10	7.90E-10
AL645608.7	2.109676	3.116449	7.72E-16	8.14E-15
AC112251.1	2.104526	3.495838	0.001840406	0.00376836
AC015712.7	2.098663	2.922411	2.45E-16	2.74E-15
AC093627.7	2.095436	3.637634	1.18E-08	5.28E-08
LINC01978	2.094611	3.373674	3.22E-15	3.19E-14
LINC00656	2.093773	2.26857	1.43E-09	7.25E-09
LINC02266	2.093392	2.234554	3.49E-12	2.46E-11
MYOSLID	2.092829	4.105667	1.03E-11	6.84E-11
LINC00589	2.084749	3.046116	1.11E-14	1.04E-13
AL135903.2	2.08087	4.062064	6.50E-17	7.79E-16
AC007881.2	2.077488	2.253113	7.76E-06	2.34E-05
AC064805.2	2.077099	3.007065	1.58E-09	7.99E-09
AL390778.1	2.074946	2.711229	2.29E-10	1.28E-09
LINC01645	2.07147	2.608813	6.54E-09	3.05E-08
AC005264.1	2.070282	3.626989	5.56E-26	1.82E-24
AC004233.3	2.070103	8.927796	1.49E-31	8.34E-30
LINC00942	2.068742	5.077105	2.61E-19	4.04E-18
AC107294.3	2.062809	5.718249	1.60E-27	6.14E-26
AC113386.1	2.059294	2.299438	2.17E-07	8.24E-07
AP000695.2	2.059	3.446479	2.94E-15	2.93E-14
AC090092.1	2.055465	4.976832	5.80E-20	9.70E-19
AL158206.1	2.053418	9.815686	1.88E-62	2.86E-59
LINC01615	2.035864	4.682284	4.76E-18	6.42E-17
AC083967.1	2.031586	2.16012	0.000139714	0.00034715
AC087491.1	2.023615	2.705675	1.67E-15	1.72E-14
AC015712.1	2.015319	5.578147	7.44E-26	2.38E-24
AC110618.1	2.01481	3.848164	2.21E-13	1.79E-12
ARAP1-AS1	2.014587	2.345651	0.000139607	0.000347
OR7E47P	2.014146	3.516395	8.00E-13	6.05E-12
AL079303.2	2.009464	6.499695	8.82E-18	1.17E-16
BX255925.1	2.002706	5.140453	4.94E-31	2.73E-29
AC084083.1	1.995267	3.414966	3.25E-16	3.56E-15
LINC01738	1.989424	2.456624	3.49E-07	1.28E-06
BX255923.1	1.982789	2.825984	0.000445453	0.00101527
AC011503.1	1.977839	2.933686	1.67E-16	1.89E-15
AC022392.1	1.976157	2.390976	5.95E-08	2.43E-07
AC046143.1	1.975069	6.364754	1.53E-42	2.97E-40

AC239803.2	1.967815	2.450603	7.73E-08	3.11E-07
AL034399.2	1.964418	3.658867	7.62E-19	1.11E-17
LINC01230	1.963949	2.031827	7.69E-08	3.10E-07
AC009336.1	1.963535	2.430429	3.38E-09	1.64E-08
BX284668.2	1.960598	4.780913	1.67E-11	1.08E-10
AL356218.1	1.94156	2.213764	0.004820608	0.00908707
TTLL11-IT1	1.937628	2.870835	9.21E-13	6.91E-12
SPATA3-AS1	1.935655	3.145155	1.16E-06	3.98E-06
AL499627.2	1.933037	2.527086	0.001101081	0.00234518
AL031985.3	1.932145	8.261423	2.74E-43	5.62E-41
AC022509.1	1.930981	3.255067	2.09E-09	1.04E-08
U62317.1	1.93037	5.865476	5.80E-07	2.07E-06
FP671120.3	1.92933	3.317035	0.001399155	0.0029333
LINC00520	1.928763	2.60674	2.30E-08	9.82E-08
LINC01655	1.928213	2.373976	5.85E-06	1.80E-05
AC107294.1	1.927422	4.289271	7.89E-20	1.31E-18
GLIS3-AS1	1.925021	5.856359	2.00E-08	8.62E-08
AC092894.1	1.924469	2.385694	0.000757558	0.00166548
AL137230.1	1.923987	2.537394	1.25E-05	3.66E-05
AC125611.4	1.92172	3.749809	1.14E-13	9.58E-13
AC022784.1	1.917697	4.744599	9.76E-17	1.14E-15
AC012213.1	1.913476	5.222766	1.96E-14	1.78E-13
LINC00941	1.905073	5.086046	2.18E-15	2.20E-14
AC090409.1	1.904974	5.651341	3.92E-26	1.31E-24
U91319.1	1.901092	2.772925	9.01E-06	2.68E-05
SH3PXD2A-AS1	1.901049	4.103429	6.59E-21	1.20E-19
LINC01451	1.889182	3.32816	3.66E-13	2.91E-12
LINC01704	1.888208	3.669317	2.78E-22	5.91E-21
MIR646HG	1.887605	7.323977	1.17E-12	8.66E-12
AC026369.2	1.885985	7.113939	1.49E-19	2.38E-18
CERS3-AS1	1.883364	3.234753	3.87E-07	1.42E-06
EGOT	1.881109	6.001307	1.50E-14	1.38E-13
AL078594.1	1.877204	2.193271	1.93E-09	9.68E-09
AC019117.1	1.872063	3.660797	1.55E-11	1.01E-10
AL138690.1	1.870517	2.730444	3.08E-10	1.69E-09
GAPLINC	1.867809	4.752998	2.99E-23	6.90E-22
AL034346.1	1.863982	3.747328	0.000136341	0.00033978
LINC01759	1.862343	7.30022	8.11E-62	1.02E-58
MIR34AHG	1.860011	3.912073	5.80E-40	8.29E-38
AC012507.2	1.859739	2.279255	6.68E-05	0.00017553
AC015712.6	1.859603	4.256131	1.44E-12	1.06E-11
AC092118.1	1.857815	6.969338	8.82E-25	2.54E-23
P4HA2-AS1	1.856337	3.179595	2.89E-24	7.52E-23
FOXD2-AS1	1.852922	6.996756	5.01E-35	3.99E-33
AC055717.1	1.852411	2.028607	0.000204587	0.00049221
AC104574.1	1.851802	1.975688	0.000107465	0.00027157
AC245100.7	1.847895	4.187394	4.02E-10	2.18E-09
AL390778.2	1.847588	3.319365	2.75E-09	1.35E-08
AC079760.2	1.845764	4.703209	8.68E-13	6.52E-12
AC007750.1	1.843932	3.641027	7.35E-13	5.61E-12
AC025154.2	1.843813	6.881095	4.01E-13	3.18E-12
AC009229.3	1.842614	2.51022	2.83E-12	2.01E-11
AC115099.1	1.842315	3.478303	4.54E-11	2.77E-10
MAPT-IT1	1.835844	2.039974	0.000943015	0.00203425
AL117372.1	1.834327	2.380466	3.59E-05	9.83E-05
C20orf166-AS1	1.833859	4.442319	0.000250299	0.000595
AL162431.1	1.831728	3.085847	8.99E-12	6.02E-11
SNAP25-AS1	1.831075	4.481573	1.76E-16	1.99E-15

AC006566.1	1.829571	5.21494	4.75E-37	5.00E-35
LINC02442	1.828574	2.022702	6.03E-09	2.83E-08
AC078864.1	1.826339	6.39868	1.86E-17	2.37E-16
AL450998.2	1.820682	2.916166	5.31E-14	4.61E-13
LINC00856	1.815702	3.539906	1.10E-08	4.95E-08
AC112229.4	1.815165	2.109082	1.02E-07	4.05E-07
AC234775.3	1.814514	6.784145	4.59E-15	4.50E-14
AC008840.1	1.810354	4.783008	1.12E-34	8.71E-33
AL391832.3	1.809636	3.586529	6.97E-19	1.03E-17
AP001995.1	1.809101	2.491908	2.30E-12	1.65E-11
Z82214.1	1.804375	3.314931	1.46E-08	6.43E-08
AP001628.2	1.801398	2.567412	2.60E-07	9.75E-07
AC093323.2	1.799952	6.320398	2.65E-16	2.94E-15
AL445224.1	1.799544	3.201381	7.47E-06	2.26E-05
LINC01968	1.799101	4.391564	7.66E-16	8.10E-15
LINC00491	1.79468	2.182263	0.00806919	0.01453106
FAM83A-AS1	1.794624	4.38597	8.25E-08	3.31E-07
AC011284.1	1.793879	2.030872	3.36E-07	1.24E-06
AC079600.3	1.786345	2.252492	4.10E-07	1.50E-06
AL645608.8	1.785162	6.408418	6.64E-22	1.35E-20
AL356752.1	1.776904	2.224453	5.17E-13	4.03E-12
AC022509.2	1.77579	6.241877	4.57E-36	4.28E-34
AC005330.1	1.775453	3.796927	7.15E-12	4.85E-11
AP000345.3	1.775185	6.511771	1.63E-24	4.50E-23
AC005041.2	1.775182	4.756679	5.61E-29	2.62E-27
AC108751.4	1.77139	2.874448	9.00E-08	3.60E-07
AP001626.1	1.770956	4.748426	2.07E-19	3.27E-18
LEMD1-AS1	1.768744	4.859037	1.91E-24	5.20E-23
LINC01122	1.768079	5.201438	2.18E-15	2.21E-14
AL359694.2	1.766185	2.808333	2.23E-09	1.10E-08
AC087286.1	1.764858	3.252393	9.18E-06	2.73E-05
AC129507.4	1.763336	3.768756	6.78E-09	3.15E-08
AC093904.4	1.762805	2.127545	1.47E-05	4.25E-05
AL022316.1	1.751335	4.302685	3.89E-16	4.21E-15
AC015849.1	1.750795	3.790132	2.32E-09	1.15E-08
IL12A-AS1	1.74977	4.224782	2.76E-10	1.52E-09
AC022784.6	1.743689	2.494184	4.46E-10	2.40E-09
AF131215.7	1.743478	7.875992	3.82E-24	9.71E-23
HOXC-AS1	1.741638	2.511737	2.75E-05	7.66E-05
AP003716.1	1.739959	4.94161	3.83E-07	1.40E-06
AL391832.2	1.737485	4.788947	2.33E-20	4.05E-19
AL358613.1	1.736817	2.046653	2.31E-06	7.55E-06
ZNF571-AS1	1.732714	7.882652	2.09E-24	5.63E-23
AP000797.3	1.730536	2.992744	2.16E-12	1.55E-11
AC078909.2	1.727643	2.278416	2.29E-06	7.50E-06
LINC00578	1.725679	5.082591	5.06E-12	3.48E-11
AL358613.2	1.719813	2.041028	8.27E-08	3.31E-07
AC009093.1	1.718139	4.500404	3.92E-09	1.88E-08
LINC01671	1.71715	3.065262	1.50E-07	5.83E-07
AC109309.1	1.716536	6.885168	2.61E-14	2.33E-13
AC026471.3	1.715482	5.919886	1.98E-24	5.37E-23
C8orf34-AS1	1.708992	4.440284	2.14E-10	1.20E-09
AC007614.2	1.707578	2.031094	3.16E-06	1.01E-05
AC024909.1	1.707187	2.202876	1.99E-06	6.58E-06
AL391244.2	1.70683	4.394509	7.61E-23	1.72E-21
LBX2-AS1	1.706106	8.197204	6.44E-33	4.21E-31
AC025176.1	1.703665	2.704062	5.20E-11	3.14E-10
AC010904.2	1.700724	3.067826	1.00E-16	1.17E-15

CASC15	1.699789	7.364128	2.70E-18	3.71E-17
AL020998.1	1.698997	1.992849	0.000174584	0.00042638
LINC01224	1.695738	5.256212	6.30E-19	9.32E-18
AC105411.1	1.694221	4.124372	4.36E-11	2.67E-10
AC131254.1	1.693292	2.995722	2.85E-08	1.21E-07
CD44-AS1	1.692811	5.619003	1.56E-20	2.74E-19
AC242842.1	1.690037	6.138626	1.62E-12	1.18E-11
AC044810.2	1.687429	2.104017	4.51E-05	0.00012137
AP005328.1	1.683953	2.621889	0.004073065	0.00780808
AC084816.1	1.683931	2.160046	0.000306855	0.00071951
BARX1-AS1	1.68331	2.374638	0.003232628	0.0063201
LINC01479	1.681837	2.507217	1.08E-10	6.23E-10
AC021242.3	1.674168	6.895362	6.75E-17	8.07E-16
LINC02115	1.669434	2.430133	1.21E-11	7.96E-11
RNF144A-AS1	1.669151	4.243763	6.84E-09	3.18E-08
AC007406.3	1.662704	6.22157	4.67E-17	5.70E-16
AC117513.1	1.661974	2.047396	9.86E-10	5.10E-09
AC127164.1	1.659688	5.0302	1.96E-16	2.21E-15
AC087612.1	1.659024	2.270814	1.15E-07	4.53E-07
AP001258.1	1.656358	10.3561	4.30E-28	1.74E-26
TDRKH-AS1	1.656134	6.645388	5.47E-48	1.80E-45
RARA-AS1	1.650802	8.967261	8.96E-47	2.51E-44
AL121985.1	1.649845	4.984886	4.84E-06	1.52E-05
AC017033.1	1.646408	2.208053	7.27E-06	2.21E-05
LINC01823	1.641187	2.241759	7.54E-06	2.28E-05
AL078590.3	1.641135	4.428181	1.14E-23	2.76E-22
MIR3945HG	1.638065	4.079608	7.21E-07	2.55E-06
AC005821.1	1.638042	2.714255	8.26E-10	4.33E-09
AC090204.1	1.637485	7.836532	5.17E-21	9.50E-20
SLC6A1-AS1	1.636722	2.219393	7.00E-07	2.48E-06
AC092167.1	1.630092	2.444047	1.18E-08	5.27E-08
AC105285.1	1.625483	7.932632	2.16E-40	3.20E-38
AL160408.3	1.625132	2.93663	1.12E-06	3.84E-06
AL596442.2	1.624494	4.638112	3.38E-20	5.79E-19
AC104794.4	1.623698	5.351146	2.04E-06	6.74E-06
AC008514.1	1.623178	3.22841	1.85E-14	1.69E-13
AC007362.1	1.620001	3.541529	2.85E-10	1.57E-09
LINC01842	1.616696	2.413404	4.04E-10	2.19E-09
AC090409.2	1.616182	2.090378	4.00E-08	1.67E-07
AC116337.3	1.60838	2.284393	9.57E-09	4.33E-08
AL662890.2	1.608353	3.582386	1.40E-16	1.61E-15
LINC00242	1.60335	6.043169	3.65E-12	2.56E-11
AL157394.1	1.60133	6.56246	7.58E-33	4.78E-31
AC009139.1	1.600529	2.278137	7.31E-08	2.95E-07
AC024909.3	1.600214	7.167445	2.12E-14	1.91E-13
PACERR	1.600063	4.654083	1.55E-14	1.43E-13
C10orf91	1.597561	5.078467	1.37E-14	1.26E-13
AL589765.6	1.597102	4.352835	1.57E-22	3.40E-21
AC003985.2	1.591334	3.104548	0.021636783	0.03569692
LINC00574	1.590656	4.658374	7.87E-11	4.65E-10
AL390036.1	1.585421	4.088828	5.14E-08	2.11E-07
AC068987.4	1.571651	7.074821	2.05E-16	2.32E-15
SPANXA2-OT1	1.569009	4.268373	7.44E-16	7.91E-15
LINC01929	1.567644	4.349113	2.30E-10	1.28E-09
LINP1	1.565605	4.066058	1.85E-07	7.09E-07
AL022315.1	1.565222	2.301837	2.41E-08	1.03E-07
AL662860.1	1.564591	2.023904	7.59E-05	0.00019769
AL022318.1	1.55927	4.077422	8.44E-13	6.35E-12

MIR222HG	1.557948	10.50865	1.24E-14	1.15E-13
AC131157.1	1.556124	2.306898	0.003714351	0.00717489
BLACAT1	1.555541	9.453129	7.81E-36	6.96E-34
AC103949.1	1.552791	1.966728	2.55E-07	9.59E-07
MIRLET7DHG	1.551105	4.656963	1.45E-24	4.01E-23
AL136964.1	1.550702	3.292395	2.06E-07	7.87E-07
Z99943.1	1.548417	3.273127	3.61E-12	2.54E-11
AC093627.2	1.547206	2.68947	1.45E-05	4.20E-05
AC243830.1	1.546863	2.910145	3.07E-11	1.92E-10
AC022784.8	1.545178	5.198244	7.42E-16	7.90E-15
MUC19	1.544025	3.115759	1.06E-08	4.76E-08
AP003498.1	1.543181	2.323741	1.06E-09	5.49E-09
AP003555.2	1.541741	4.341349	1.33E-15	1.37E-14
Z82186.1	1.539905	4.769428	2.46E-09	1.21E-08
AC100793.3	1.539669	2.850543	2.22E-10	1.24E-09
AL139161.1	1.538862	3.709677	2.88E-12	2.04E-11
AC090578.1	1.537443	2.411783	2.67E-07	1.00E-06
AP003555.3	1.536394	2.720912	3.73E-10	2.03E-09
AL390729.1	1.535592	3.534618	4.47E-10	2.41E-09
LINC01843	1.533382	3.388788	4.88E-11	2.96E-10
LINC01559	1.528154	2.283167	8.39E-06	2.51E-05
AC079380.1	1.525994	3.324314	1.88E-12	1.36E-11
AL356124.1	1.524567	3.305082	1.33E-17	1.71E-16
MUC2	1.524352	2.121069	1.89E-05	5.37E-05
UPP2-IT1	1.52416	2.420294	7.16E-11	4.25E-10
LINC01010	1.524078	4.809562	8.32E-07	2.92E-06
AL590004.4	1.520894	6.77517	5.80E-07	2.07E-06
LINC01949	1.520847	2.442633	1.00E-07	3.98E-07
AC087588.2	1.520354	2.419908	5.27E-06	1.64E-05
AC068051.1	1.518521	2.129676	1.99E-07	7.59E-07
AC015914.1	1.515958	7.511647	6.82E-13	5.22E-12
AC002384.1	1.514731	2.371974	0.00010259	0.00025985
AL158151.4	1.513811	3.282272	2.81E-12	2.00E-11
LINC00677	1.51348	2.682179	2.94E-09	1.43E-08
AL353803.5	1.512623	2.172622	6.54E-08	2.65E-07
LINC01546	1.511227	2.987717	8.87E-15	8.43E-14
AC011352.3	1.507102	2.046164	0.000341767	0.00079522
AL158152.2	1.506253	5.870343	1.82E-29	8.82E-28
AC015983.2	-1.50117	2.387055	1.03E-11	6.84E-11
LINC01877	-1.50496	2.729107	4.17E-17	5.14E-16
CYP17A1-AS1	-1.50667	3.329767	1.54E-24	4.25E-23
NAMA	-1.51108	3.517106	8.47E-22	1.68E-20
AC110491.1	-1.51755	3.15948	2.18E-14	1.96E-13
AC098818.2	-1.51849	2.806931	1.39E-15	1.44E-14
AC011700.1	-1.52015	4.750651	7.05E-14	6.04E-13
AC019211.1	-1.52083	3.204555	5.11E-12	3.51E-11
AC106028.5	-1.52524	2.055379	5.38E-14	4.65E-13
AC010735.1	-1.526	3.789283	5.67E-17	6.85E-16
LINC02097	-1.52706	2.728873	1.71E-15	1.75E-14
LINC01361	-1.52713	3.198941	8.32E-25	2.41E-23
AC125618.1	-1.53142	2.682195	7.05E-17	8.41E-16
NAV2-AS4	-1.53362	3.375814	2.68E-15	2.69E-14
AC069281.1	-1.53719	1.985087	9.12E-10	4.74E-09
PGM5-AS1	-1.53755	3.395344	5.19E-15	5.05E-14
AC120498.1	-1.53806	3.906847	1.30E-11	8.53E-11
HAND2-AS1	-1.53893	5.292555	2.01E-13	1.64E-12
AL928768.1	-1.5394	4.340533	1.84E-05	5.25E-05
AL449403.1	-1.54403	2.27454	6.03E-13	4.66E-12

AC007365.1	-1.54718	4.277333	1.60E-36	1.63E-34
LINC00668	-1.55114	2.27941	4.80E-05	0.00012855
LINC01405	-1.55236	2.148338	0.000459537	0.00104642
LINC00926	-1.5543	7.178441	8.24E-18	1.09E-16
AC093903.1	-1.55442	2.408593	8.20E-07	2.88E-06
DIO3OS	-1.55543	5.367642	9.63E-10	4.99E-09
AC025279.1	-1.55749	4.079943	1.91E-17	2.42E-16
AC103957.2	-1.55803	5.349849	4.38E-17	5.37E-16
AC008268.1	-1.55859	3.732734	3.09E-16	3.39E-15
LINC01754	-1.56014	2.188162	7.96E-06	2.40E-05
AC093843.1	-1.56611	2.781733	1.92E-10	1.08E-09
ATP6V0E2-AS1	-1.56689	8.358191	3.60E-26	1.22E-24
AC007563.2	-1.56852	2.76386	4.43E-30	2.24E-28
AC104971.4	-1.57289	2.85632	4.15E-06	1.31E-05
AC253536.3	-1.57463	4.546376	3.40E-22	7.20E-21
LINC01564	-1.57768	2.553096	5.47E-11	3.29E-10
AC002546.2	-1.57851	2.987377	1.96E-08	8.48E-08
AC084880.3	-1.58051	2.468282	5.62E-11	3.38E-10
AL359853.1	-1.58129	3.925148	1.07E-08	4.83E-08
AL355916.2	-1.58225	5.323848	2.56E-24	6.73E-23
AC008440.3	-1.58888	3.341565	1.57E-21	3.07E-20
AL157902.1	-1.59107	2.168605	1.03E-20	1.85E-19
AC092068.1	-1.59913	2.895611	1.57E-12	1.15E-11
AC132938.1	-1.59979	4.823604	9.38E-18	1.23E-16
AL049874.3	-1.60294	2.191982	9.41E-14	7.96E-13
AL161668.3	-1.60574	2.138013	2.60E-16	2.89E-15
AC106795.5	-1.60661	2.740569	8.91E-15	8.46E-14
AC036108.1	-1.61419	2.953762	1.37E-26	4.78E-25
AC093607.1	-1.61844	1.986474	1.15E-08	5.17E-08
AP001178.3	-1.61941	4.442576	1.07E-34	8.43E-33
AC093797.1	-1.61977	6.478633	5.28E-21	9.69E-20
AC025259.3	-1.62443	7.081235	8.11E-22	1.62E-20
AL353803.4	-1.62464	2.439475	9.14E-10	4.75E-09
HSD17B3-AS1	-1.62692	2.510648	6.87E-20	1.15E-18
AP000721.2	-1.63062	3.219167	3.54E-12	2.49E-11
AC008659.1	-1.63441	2.098611	3.78E-17	4.69E-16
AC069208.1	-1.63879	3.439293	3.72E-27	1.38E-25
LINC02242	-1.64328	2.137016	4.10E-08	1.71E-07
PRICKLE2-AS1	-1.64442	2.961317	9.84E-23	2.19E-21
NCAM1-AS1	-1.64903	3.326227	5.18E-15	5.05E-14
FER1L6-AS2	-1.65414	1.998098	2.29E-15	2.31E-14
TPRG1-AS1	-1.65471	3.910345	6.10E-23	1.38E-21
AC073130.1	-1.65653	3.79521	3.52E-21	6.56E-20
AC091078.1	-1.65982	2.01731	4.07E-07	1.49E-06
AC104232.1	-1.66029	3.285494	1.19E-34	9.19E-33
AC078923.1	-1.66683	3.323547	2.87E-15	2.86E-14
AL445426.1	-1.667	2.850336	4.48E-18	6.05E-17
AC025280.1	-1.67208	2.018461	1.14E-17	1.47E-16
AC005086.1	-1.68437	2.321067	9.04E-23	2.02E-21
AL355483.3	-1.6849	2.487729	1.42E-11	9.29E-11
AC100823.1	-1.68583	2.28475	8.36E-10	4.38E-09
AC108472.1	-1.68749	3.147262	5.52E-18	7.42E-17
AC134682.1	-1.69718	3.874528	9.02E-23	2.02E-21
AL158210.1	-1.70592	2.385916	5.25E-16	5.62E-15
LINC02276	-1.70746	3.054437	3.12E-20	5.36E-19
SMAD9-IT1	-1.70856	4.233964	4.42E-28	1.78E-26
AC013264.1	-1.71195	3.08083	1.72E-11	1.11E-10
AL392089.1	-1.71762	1.997759	2.68E-16	2.97E-15

AF067845.1	-1.723	3.743186	7.79E-11	4.61E-10
AC245014.3	-1.72302	4.318812	5.56E-53	3.24E-50
AC002306.1	-1.72479	2.87112	9.56E-27	3.37E-25
AC106795.3	-1.72766	2.672224	5.76E-13	4.46E-12
TCL6	-1.7279	4.49753	1.00E-07	4.00E-07
AC107029.2	-1.73059	2.466126	4.90E-24	1.22E-22
AP000757.1	-1.73176	5.223417	2.89E-23	6.73E-22
AL360268.1	-1.75153	1.943592	5.71E-18	7.64E-17
AC002511.2	-1.75219	3.344704	1.45E-15	1.50E-14
FAM95C	-1.75289	6.552177	1.26E-24	3.52E-23
TRHDE-AS1	-1.76111	2.869069	9.23E-17	1.08E-15
LINC01722	-1.77037	2.040614	7.49E-16	7.95E-15
AC010857.1	-1.77084	4.689791	3.05E-07	1.13E-06
AL160286.2	-1.77085	2.669713	4.72E-25	1.40E-23
SYNE1-AS1	-1.77201	3.343684	5.53E-25	1.62E-23
AL035425.1	-1.78234	6.292514	1.90E-20	3.32E-19
AC022239.4	-1.79011	2.527473	1.40E-07	5.44E-07
AL157911.1	-1.79944	2.541003	4.92E-26	1.62E-24
AC022239.2	-1.81085	3.756973	9.14E-11	5.34E-10
MIR3681HG	-1.81406	2.798312	3.71E-12	2.60E-11
AL590302.2	-1.81527	2.09688	1.32E-08	5.84E-08
TERC	-1.81531	2.113985	2.21E-22	4.78E-21
AC099792.1	-1.81654	2.47009	5.63E-20	9.44E-19
LINC00402	-1.82903	3.97078	6.18E-08	2.52E-07
AC010207.1	-1.83209	5.182217	2.39E-39	3.18E-37
AC007204.1	-1.84005	7.583414	3.53E-21	6.56E-20
AC007998.3	-1.85263	4.442322	2.15E-33	1.43E-31
AL050338.2	-1.85467	2.657325	2.37E-16	2.66E-15
LINC01781	-1.86101	4.092666	2.18E-07	8.28E-07
AC008083.1	-1.86643	2.309264	2.13E-09	1.06E-08
AC007221.1	-1.86973	2.428209	5.75E-13	4.45E-12
AC022239.3	-1.87075	2.764648	1.44E-08	6.37E-08
AC105053.1	-1.87131	4.828641	3.69E-36	3.54E-34
LINC02397	-1.88205	4.328665	1.76E-11	1.13E-10
LINC02432	-1.88847	6.317861	5.55E-36	5.13E-34
LINC02413	-1.89432	2.446891	8.86E-11	5.18E-10
AC078820.1	-1.89443	2.730174	6.21E-19	9.21E-18
AC008649.2	-1.91708	1.97816	3.76E-22	7.92E-21
AC090541.1	-1.91894	2.517984	1.04E-12	7.75E-12
AL512631.1	-1.93823	3.210288	5.68E-09	2.67E-08
LINC01354	-1.93898	5.593141	8.98E-31	4.89E-29
AL121759.1	-1.94117	2.061072	3.00E-28	1.26E-26
LINC02301	-1.96796	2.543794	1.65E-08	7.24E-08
AL161781.2	-1.96875	2.376744	5.78E-07	2.07E-06
AC023590.1	-1.97347	4.075396	1.82E-24	4.97E-23
AC022239.1	-1.97877	2.401888	1.02E-09	5.27E-09
AC007381.1	-1.97921	2.528746	8.43E-15	8.05E-14
C12orf77	-1.98014	2.214778	3.07E-11	1.92E-10
AC069234.1	-1.98608	2.442126	1.34E-18	1.89E-17
AP000936.1	-1.98683	1.958491	4.97E-21	9.17E-20
LINC01659	-1.9923	3.137504	3.80E-20	6.48E-19
AC022217.2	-2.0078	2.857269	3.16E-33	2.08E-31
AC148477.3	-2.01212	4.118184	8.02E-17	9.48E-16
AL158829.1	-2.01222	2.627326	4.94E-15	4.82E-14
AL591501.1	-2.01397	2.554223	3.61E-16	3.93E-15
FAM167A-AS1	-2.01978	4.776233	7.84E-13	5.93E-12
AL390726.3	-2.02141	2.346978	1.25E-29	6.12E-28
AP001271.1	-2.02154	2.552276	1.14E-28	5.07E-27

LINC01519	-2.02723	2.582378	2.90E-16	3.19E-15
AC008440.1	-2.02839	4.154632	8.69E-44	1.99E-41
AC148477.2	-2.03182	5.642104	2.16E-21	4.14E-20
AC108058.1	-2.03796	3.981266	4.38E-30	2.23E-28
AC097059.1	-2.04704	3.275263	2.57E-19	3.98E-18
AP002518.2	-2.04917	2.000643	4.24E-17	5.23E-16
LINC01502	-2.05207	4.881033	4.05E-09	1.94E-08
LINC01991	-2.05692	2.677438	3.06E-10	1.68E-09
FGF10-AS1	-2.05806	1.997679	1.28E-14	1.19E-13
AC141930.1	-2.0637	5.794531	2.23E-11	1.42E-10
LINC01257	-2.06979	4.641616	3.78E-12	2.65E-11
MIR663AHG	-2.07108	2.147562	4.55E-10	2.45E-09
AC006305.3	-2.08222	3.465008	4.97E-24	1.23E-22
AC062015.1	-2.09802	8.264557	1.02E-17	1.33E-16
LINC01589	-2.09874	2.288311	1.07E-18	1.53E-17
AL359815.1	-2.1043	2.515949	3.46E-18	4.71E-17
AC004877.1	-2.10963	4.881671	7.96E-33	4.98E-31
AC004832.1	-2.12111	2.046249	1.28E-18	1.82E-17
AL162727.1	-2.12552	2.627161	9.48E-30	4.69E-28
AP000802.1	-2.12763	4.595085	3.45E-24	8.83E-23
LINC02132	-2.13241	3.001442	1.84E-15	1.88E-14
AL355102.1	-2.13244	2.509197	1.27E-19	2.04E-18
AL353746.1	-2.13362	5.889905	5.63E-13	4.36E-12
AC008676.2	-2.14222	3.48452	8.79E-36	7.74E-34
AC078842.1	-2.1438	4.050277	6.45E-22	1.32E-20
AL139020.1	-2.15246	3.591101	1.65E-07	6.37E-07
AC002511.1	-2.16377	3.555258	2.37E-25	7.39E-24
AC092127.2	-2.17199	3.460184	1.66E-35	1.43E-33
LINC02308	-2.17321	3.34005	1.21E-18	1.72E-17
LINC01695	-2.17737	2.619227	6.06E-31	3.33E-29
AL596247.1	-2.20387	2.76566	1.17E-24	3.29E-23
AC093609.1	-2.22008	4.110265	5.82E-43	1.16E-40
AC116345.3	-2.23347	2.220468	2.32E-18	3.21E-17
AC026904.2	-2.2529	2.344862	1.83E-11	1.17E-10
AC026116.1	-2.25311	5.161196	1.06E-10	6.11E-10
AL359915.1	-2.27526	2.635104	1.90E-27	7.24E-26
AL138767.3	-2.27841	3.008952	5.74E-26	1.87E-24
LINC01672	-2.30407	3.63787	7.20E-33	4.63E-31
AP002370.2	-2.30463	2.74462	5.11E-27	1.86E-25
AC016573.1	-2.3059	2.600148	2.54E-21	4.84E-20
AC016168.2	-2.31134	1.992704	1.77E-12	1.29E-11
LINC00602	-2.31611	2.963762	1.12E-19	1.83E-18
AC079062.1	-2.31764	2.487643	7.97E-24	1.96E-22
AC108206.1	-2.3256	3.455508	1.22E-10	7.02E-10
AL590867.1	-2.3454	2.914722	2.95E-15	2.94E-14
LINC01975	-2.34622	4.013656	2.58E-16	2.88E-15
AP003548.1	-2.35795	2.857547	3.29E-31	1.83E-29
AC007091.1	-2.37597	2.270011	1.64E-15	1.68E-14
LINC01539	-2.38314	5.52814	3.73E-35	3.00E-33
MIR4500HG	-2.39015	2.421466	9.25E-19	1.33E-17
AL133346.1	-2.39677	3.885213	7.49E-40	1.03E-37
EPHA5-AS1	-2.42072	4.043369	8.94E-14	7.61E-13
AC006305.2	-2.45818	2.786629	5.93E-30	2.98E-28
AL513303.1	-2.459	2.159816	2.85E-28	1.21E-26
AP000842.2	-2.46061	2.300843	5.11E-27	1.86E-25
AC002383.1	-2.46195	2.491156	1.65E-19	2.61E-18
LINC01532	-2.4641	2.753703	2.37E-28	1.01E-26
LINC01658	-2.47602	2.320979	5.01E-24	1.24E-22

AC110296.1	-2.50307	2.055579	4.82E-25	1.42E-23
PCAT14	-2.51028	5.231761	2.13E-25	6.70E-24
AC006305.1	-2.52513	5.900611	8.32E-38	9.40E-36
AC093010.2	-2.53331	3.447676	7.33E-25	2.13E-23
AL008628.1	-2.54169	3.30685	3.65E-41	5.98E-39
AC104137.1	-2.54914	3.249945	6.15E-14	5.30E-13
LINC01501	-2.55986	2.691102	2.44E-12	1.74E-11
LINC01765	-2.56191	2.004746	3.68E-26	1.24E-24
LINC01905	-2.59525	2.429988	6.78E-34	4.75E-32
AL136088.1	-2.60204	5.259965	2.60E-24	6.83E-23
AADACL2-AS1	-2.61571	2.138846	3.33E-28	1.39E-26
LINC01697	-2.61619	2.460499	3.41E-32	2.03E-30
AC008632.1	-2.64574	2.45278	4.38E-25	1.30E-23
AC104985.1	-2.67936	2.517988	3.67E-24	9.36E-23
AC117500.2	-2.71578	3.084189	3.54E-28	1.46E-26
AC007952.4	-2.74389	2.938923	6.04E-49	2.18E-46
AC068672.2	-2.84338	1.986933	2.42E-17	3.05E-16
AL034374.2	-2.85211	5.383087	9.00E-113	6.81E-109
AC010425.1	-2.95856	3.135387	2.38E-27	8.92E-26
AC046195.1	-2.99379	4.51468	9.69E-25	2.77E-23
LINC00473	-2.9978	7.477778	3.53E-28	1.46E-26
AF111167.1	-3.02547	2.677265	1.03E-58	8.71E-56
AL353699.1	-3.04009	2.596126	5.91E-40	8.29E-38
AC005906.2	-3.05081	2.429402	2.25E-24	6.01E-23
LINC01384	-3.05705	4.960313	8.46E-30	4.22E-28
LINC01785	-3.1391	2.61288	7.56E-22	1.51E-20
AC137056.1	-3.31837	2.014503	7.05E-61	7.64E-58
LINC01834	-3.63482	2.290721	1.86E-33	1.26E-31
Z93241.1	-3.63652	3.644426	2.18E-95	8.26E-92
