# Supplementary material for: A Potential Four-Gene Signature and Nomogram for Predicting the Overall Survival of Papillary Thyroid Cancer
Source: Dis Markers. 2022 Aug 30;2022:8735551. doi: 10.1155/2022/8735551 (PMC9526076; doi:10.1155/2022/8735551)
Supplement: Supplementary 2 — Table S1: details of the GEO and TCGA datasets used in this study. Table S2: samples in HPA database. Table S3: the sequences of primers. Table S4: univariate Cox regression of the 176 genes in the training cohort. Table S5: 96 DEmiRNAs between PTC and normal thyroid tissues. Table S6: 839 DEIncRNAs between PTC and normal thyroid tissues. Table S7: the IncRNAs, mARNAs, and miRNAs in the ceRNA network. [file 8735551.f2.zip › Table S7 (1).pdf]

Table S7. The lncRNAs, mRNAs, and miRNAs in the ceRNA network.

lncRNA	miRNA	miRNA	mRNA
IGF2-AS	hsa-mir-503	hsa-mir-503	TENM1
IGF2-AS	hsa-mir-519d	hsa-mir-503	DPT
C20orf166-AS1	hsa-mir-373	hsa-mir-519d	TENM1
C20orf166-AS1	hsa-mir-519d	hsa-mir-373	MRO
C20orf166-AS1	hsa-mir-205	hsa-mir-205	TENM1
C20orf166-AS1	hsa-mir-222	hsa-mir-205	DPT
C20orf166-AS1	hsa-mir-375	hsa-mir-205	MRO
LINC00302	hsa-mir-31	hsa-mir-222	ABI3BP
LINC00302	hsa-mir-506	hsa-mir-222	MRO
C10orf91	hsa-mir-373	hsa-mir-375	ABI3BP
AC022148.1	hsa-mir-373	hsa-mir-31	MRO
AC022148.1	hsa-mir-519d	hsa-mir-506	MRO
AC022148.1	hsa-mir-205	hsa-mir-508	ABI3BP
AC022148.1	hsa-mir-508	hsa-mir-508	MRO
AC022148.1	hsa-mir-222	hsa-mir-187	MRO
AC022148.1	hsa-mir-31		
AC007362.1	hsa-mir-506		
AC007362.1	hsa-mir-375		
LINC00313	hsa-mir-503		
LINC00313	hsa-mir-373		
LINC00313	hsa-mir-187		
LINC00313	hsa-mir-205		
LINC00313	hsa-mir-31		
LINC00313	hsa-mir-375		
TCL6	hsa-mir-503		
TCL6	hsa-mir-373		
TCL6	hsa-mir-519d		
TCL6	hsa-mir-187		
TCL6	hsa-mir-205		
TCL6	hsa-mir-222		
TCL6	hsa-mir-31		
TCL6	hsa-mir-375		
BX255923.1	hsa-mir-506		
AC009093.1	hsa-mir-373		
AC009093.1	hsa-mir-519d		
AC009093.1	hsa-mir-31		
MUC2	hsa-mir-503		
MUC2	hsa-mir-373		
AC004832.1	hsa-mir-519d		
AC004832.1	hsa-mir-508		
AC004832.1	hsa-mir-31		
MUC19	hsa-mir-503		
MUC19	hsa-mir-373		
MUC19	hsa-mir-519d		
MUC19	hsa-mir-187		
MUC19	hsa-mir-205		
MUC19	hsa-mir-508		
MUC19	hsa-mir-222		
MUC19	hsa-mir-31		

MUC19	hsa-mir-375
AC002511.1	hsa-mir-519d
AC006305.1	hsa-mir-503
AC006305.1	hsa-mir-519d
AC006305.1	hsa-mir-222
AC006305.1	hsa-mir-506
AC006305.1	hsa-mir-375
AP000525.1	hsa-mir-503
AP000525.1	hsa-mir-31
UCA1	hsa-mir-506
AC010336.2	hsa-mir-373
AC010336.2	hsa-mir-519d
AC010336.2	hsa-mir-205
AC010336.2	hsa-mir-31
CLDN10-AS1	hsa-mir-508
CLDN10-AS1	hsa-mir-222
MIR181A2HG	hsa-mir-205
LINC00365	hsa-mir-519d
SFTA1P	hsa-mir-503
SFTA1P	hsa-mir-222
LINC00475	hsa-mir-503
LINC00475	hsa-mir-205
LINC00475	hsa-mir-506
C12orf77	hsa-mir-373
C12orf77	hsa-mir-519d
C12orf77	hsa-mir-222
C12orf77	hsa-mir-31
SPANXA2-OT1	hsa-mir-373
SPANXA2-OT1	hsa-mir-375
LINC00423	hsa-mir-503
LINC00423	hsa-mir-31
HOTAIR	hsa-mir-519d
HOTAIR	hsa-mir-222
HOTAIR	hsa-mir-506
HOTAIR	hsa-mir-375
HCG22	hsa-mir-508
HCG22	hsa-mir-31
HCG22	hsa-mir-506
MIR4500HG	hsa-mir-31
LINC00242	hsa-mir-503
LINC00242	hsa-mir-222
LINC00242	hsa-mir-31
MIRLET7DHG	hsa-mir-373
MIRLET7DHG	hsa-mir-205
MIRLET7DHG	hsa-mir-375
MIR205HG	hsa-mir-205
MIR205HG	hsa-mir-222
MIR205HG	hsa-mir-31
MIR205HG	hsa-mir-506
SLC6A1-AS1	hsa-mir-508
CYP1B1-AS1	hsa-mir-205

AC110491.1	hsa-mir-205
AC110491.1	hsa-mir-508
AC110491.1	hsa-mir-222
AC110491.1	hsa-mir-506
LINC00460	hsa-mir-503
LINC00460	hsa-mir-222
LINC00284	hsa-mir-503
LINC00284	hsa-mir-519d
LINC00284	hsa-mir-205
LINC00284	hsa-mir-508
LINC00284	hsa-mir-506
TPRG1-AS1	hsa-mir-373
TPRG1-AS1	hsa-mir-519d
LINC00402	hsa-mir-519d
LINC00402	hsa-mir-506
EGOT	hsa-mir-205
EGOT	hsa-mir-375
GLIS3-AS1	hsa-mir-506
P4HA2-AS1	hsa-mir-503
P4HA2-AS1	hsa-mir-205
P4HA2-AS1	hsa-mir-508
AL158206.1	hsa-mir-373
AL158206.1	hsa-mir-222
AC068594.1	hsa-mir-222
AC011383.1	hsa-mir-31
SYNPR-AS1	hsa-mir-375
PRICKLE2-AS1	hsa-mir-373
PRICKLE2-AS1	hsa-mir-519d
GDNF-AS1	hsa-mir-503
GDNF-AS1	hsa-mir-187
LINC00491	hsa-mir-222
NAV2-AS4	hsa-mir-205
OPCML-IT1	hsa-mir-503
OPCML-IT1	hsa-mir-373
OPCML-IT1	hsa-mir-519d
OPCML-IT1	hsa-mir-205
OPCML-IT1	hsa-mir-506
OPCML-IT1	hsa-mir-375
AP001029.2	hsa-mir-503
AP001029.2	hsa-mir-31
DIO3OS	hsa-mir-373
DIO3OS	hsa-mir-508
DIO3OS	hsa-mir-222
DIO3OS	hsa-mir-506
LINC00520	hsa-mir-503
LINC00520	hsa-mir-373
LINC00520	hsa-mir-519d
LINC00520	hsa-mir-205
LINC00520	hsa-mir-31
LINC00520	hsa-mir-506
LINC00520	hsa-mir-375
